# New Formyl Phloroglucinol Meroterpenoids from the Leaves of *Eucalyptus robusta*

**DOI:** 10.1038/srep39815

**Published:** 2016-12-22

**Authors:** Zhi-Chun Shang, Ming-Hua Yang, Rui-Huan Liu, Xiao-Bing Wang, Ling-Yi Kong

**Affiliations:** 1 State Key Laboratory of Natural Medicines, Department of Natural Medicinal Chemistry, China Pharmaceutical University, 24 Tong Jia Xiang, Nanjing 210009, People’ s Republic of China

## Abstract

Seven new formyl phloroglucinol meroterpenoids (FPMs), namely eucalrobusones J-P (**1**–**7**), as well as three known ones (**8**–**10**) were isolated from the leaves of *Eucalyptus robusta*. Their structures were elucidated by spectroscopic data analysis, and their absolute configurations were determined by applications of the Snatzke’s helicity rule and the electron circular dichroism (ECD) calculation. These FPMs are diverse in coupling patterns between phloroglucinol and sesquiterpenoid units, forming novel polycyclic ring systems. Compound **1** possesses a new carbon skeleton that a 1-oxaspiro[5.6]dodecane core is formed through C-14 rather than C-4 of the aromadendrane moiety. Compound **2** features a novel 6/7/5 ring-fused 6-oxabicyclo[3.2.2]nonane skeleton. Compounds **3**–**5** are rare aristolane-based FPMs. By forming different oxo bridges, compound **3** is the first sample of FPM with benzo-dihydrofuran structure, and compound **4** possesses a novel 6/6/6/6/3-fused pentacyclic skeleton. Compounds **1**, **6**, and **8** exhibited significant antifungal activities against *Candida glabrata* with MIC_50_ values of 2.57, 1.95, and 2.49 *μ*g/mL, respectively.

In recent years, phloroglucinol derivatives have been a hot research topic of natural products, not only because of their numerous bioactivities, such as anti-HIV^1^, antimicrobial^2^, and antitumor^3^ effects, but also due to their diversified skeletons with highly complex polycyclic systems and multiple chiral centers^4^,^5^,^6^. Formyl phloroglucinol meroterpenoids (FPMs) are important derivatives of phloroglucinol. Through hetero Diels-Alder reaction^6^,^7^,^8^ or nucleophilic addition^9^,^10^,^11^, phloroglucinol of FPMs could couple with different terpene moieties, mainly forming dihydropyran-fused or open-chain structures. In addition, reaction at the active sites of phloroglucinol (C-7′/C-12′/OH-3′) could also generate other coupling patterns, such as macrocarpal A^1^, eucalrobusone A^10^, and eucalyptal A^11^. In our continuous ^1^H NMR-guided isolation^10^, ten FPMs (Fig. 1) with various spiro-heterocycles and polycyclic systems were isolated from the leaves of *Eucalyptus robusta*. These FPMs feature various oxa-heterocyclic systems with five- to eight- membered rings due to different coupling patterns between phloroglucinol and sesquiterpenoid units. Within FPMs, **1** and **3** are novel oxaspiro compounds. The unprecedented 1-oxaspiro[5.6]dodecane subunit that constituted through C-10/C-14 of aromadendrane, makes **1** a new carbon skeleton; the 1-oxaspiro[4.5]decane subunit in **3** also gives the first sample of forming dihydrofuran ring between two moieties. Compound **2** is a guaiane based FPM, in which the two rare oxo bridges between C-3′/C-6 and C-10/C-11 lead to an unusual polycyclic ring system. Compounds **3**–**5** are rare aristolane-based FPMs that only two examples were reported before^12^. In addition, these natural products showed different antifungal activities against *Candida albicans* and *C. glabrata*. Compounds **1**, **6**, and **8** showed the most potent antifungal activities against *C. glabrata* with MIC_50_ values of 2.57, 1.95, and 2.49 *μ*g/mL, respectively. Herein, we report the isolation and structure elucidation of these FPMs, as well as their antifungal activities.

## Results and Discussion

### Structure elucidation

**Figure 2:**
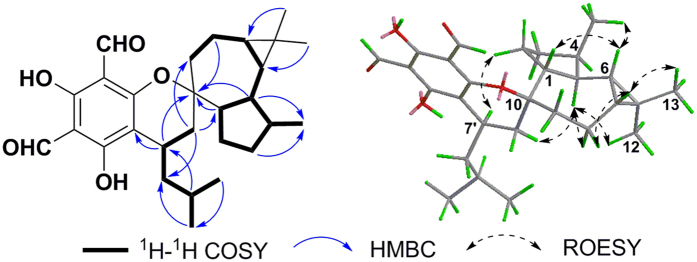
Key ^1^H-^1^H COSY, HMBC, and ROESY correlations of 1.

Eucalrobusone J (**1**) gave an HRESIMS ion at *m/z* 453.2648 [M-H]^−^, consistent with the molecular formula of C_28_H_38_O_5_. In its NMR data (Tables 1 and 2), the characteristic formyl phloroglucinol moiety was found, especially due to the chelated phenolic hydroxyls (*δ*_H_ 13.31 and 13.46, each 1H, s) and formyls (*δ*_H_ 10.03, 1H, s, *δ*_C_ 192.5 and *δ*_H_ 10.14, 1H, s, *δ*_C_ 191.9). Two secondary methyls (*δ*_H_ 0.94, 3H, d, *J* = 6.3 Hz and *δ*_H_ 0.99, 3 H, d, *J* = 6.3 Hz) and the key HMBC correlations from Me-10′ to C-11′, from Me-11′ to C-8′, and from H-9′ to C-7′ constructed an isoamyl moiety. The remaining 15 carbons that contained three methyls (*δ*_H_ 0.93, 1.02, and 1.04, each 3H) revealed a sesquiterpenoid moiety in **1**. An aromadendrane-type sesquiterpenoid moiety was established by 2D NMR analyses (Fig. 2): the ^1^H-^1^H COSY spectrum gave one spin system (C-1 to C-9) and the key HMBC correlations from Me-12 to C-6/C-7, from H_2_–3/H-5 to C-15, and from H-5/H_2_-8 to C-10 revealed the skeleton. Moreover, the obvious downfield shift of C-10 (*δ*_C_ 86.5) and the HMBC correlations from H_2_-14 to C-7′/C-8′/C-1/C-9 and from H-7′ to C-10 revealed the novel coupling pattern of two moieties, which was named the oxa-spiro [5.6] ring. The relative configuration of **1** was established by the ROESY spectrum. The key correlations (Fig. 2) of H-7′/H-2*β* and H-14*β*/H-5 indicated that H-7′ and H-2*β* were closely proximate in space and that the two planes of dihydropyran and tetrahydrooxepine rings were in a vertically approximate manner. The ROESY correlations of H-5/H-8*β*, H-7/H-8*α*, Me-12/H-5, Me-15/H-6, and H-6/H-1 indicated the *α*-orientations of H-1, H-6, and H-7 and the *β*-orientations of H-4 and H-5. The absolute configuration was determined by application of the Snatzke’s helicity rule^10^,^13^,^14^,^15^,^16^. The *P/M*-helicity of the dihydropyran ring in FPMs controlled the signs of Cotton effects approximately between 270–310 nm (Fig. 6). Therefore, the negative Cotton effect at 310 nm (Δ*ε* = −2.97, Fig. 6A) assigned the 10*S* and 7′*R* configurations.

**Figure 3:**
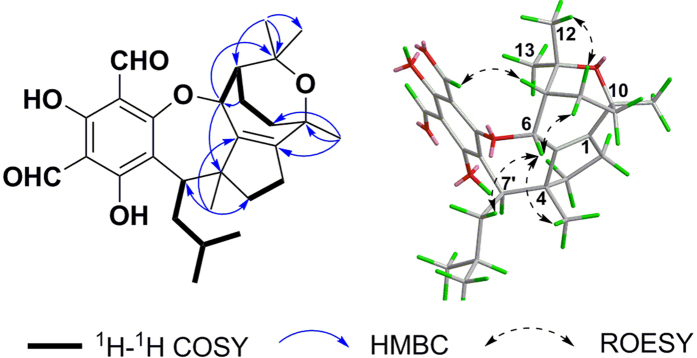
Key ^1^H-^1^H COSY, HMBC, and ROESY correlations of 2.

Eucalrobusone K (**2**), C_28_H_36_O_6_, was also a phloroglucinol-sesquiterpene adduct. The comprehensive analysis of NMR data (Tables 1 and 2) verified its modified guaiane-type moiety. The ^1^H-^1^H COSY spectrum established two spin systems (C-2 to C-3 and C-6 to C-9), and the HMBC correlations from Me-14 to C-1 and from Me-15 to C-5 located a tetrasubstituted double bond at Δ^1(5)^. The key HMBC correlations (Fig. 3) of H_2_-8 with C-6/C-10/C-11, of Me-15 with C-3/C-4/C-5/C-7′, of Me-13 with C-12/C-11/C-7, and of Me-14 with C-1/C-9/C-10 subsequently confirmed the carbon skeleton of its sesquiterpene moiety, which coupled with the isoamyl through the C-7′-C-4 bond. Meanwhile, the remaining two degrees of unsaturation and the observed four oxygenated carbons at C-3′, C-6, C-10, and C-11 (*δ*_C_ 168.4, 85.8, 71.2, and 74.6, respectively) revealed that there were two oxygen bridges in **2**. The diagnostic downfield shift of C-6 (*δ*_C_ 85.8) revealed its ether linkage to the deshielding benzene ring^10^,^11^,^17^, and consequently the oxygen bridge between C-10 and C-11 was established to fulfil the unsaturated degree^18^. The ROESY correlations (Fig. 3) of H-6/H-8′a, H-6/Me-15, H-6/H-8*β*, H-8*α*/Me-12, and H-12′/H-7 revealed the *α*-oriented H-7′ and the *β*-oriented H-6, H-7, Me-14, and Me-15. In order to determine its absolute configuration, quantum chemical electron circular dichroism (ECD) calculation was applied^10^,^17^, and indicated the 4*R*, 6*R*, 7*R*, 10*S*, and 7′*R* configurations (Figure S1).

**Figure 4:**
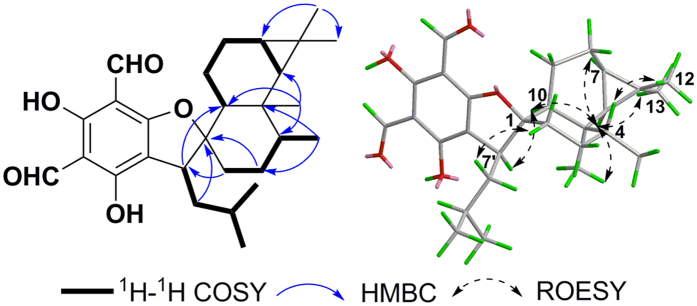
Key ^1^H-^1^H COSY, HMBC, and ROESY correlations of 3.

Eucalrobusone L (**3**) was assigned the molecular formula of C_28_H_38_O_5_ according to its pseudomolecular ion peak at *m/z* 453.2650 [M-H]^−^. Analysis of NMR data (Tables 1 and 2) also established the formyl phloroglucinol moiety. The existence of two spin systems (C-2 to C-15 and C-6 to C-10) and the key HMBC correlations from Me-12 to C-6/C-7, from Me-14 to C-4/C-6/C-10, and from H-2/H-3/H-10 to C-1 (Fig. 4) established the aristolane-type sesquiterpene moiety. The HMBC correlations from H_2_-8′ to C-1, from H-7′ to C-10, and from H_2_-2 to C-7′ established that the two moieties were coupled through the C-7′-C-1 bond. However, the two moieties only occupied nine degrees of unsaturation. The remained one degree of unsaturation and the very downfield shift of C-1 (*δ*_C_ 98.9) revealed the oxa-spiro [4.5] ring between above two moieties^10^,^11^. The two moieties were arranged in an approximately vertical manner by the key ROESY correlations (Fig. 4) of H-7′/H-10 and H-8′a/H-2*α*. Subsequently, the ROESY correlations of H-6/Me-14, H-6/Me-13, H-6/H-10, and H-4/Me-12 assigned the *β*-oriented H-4 and the *α*-oriented H-6, H-7, H-10, Me-14, and H-7′. The absolute configuration of **3** was determined as 1*R*, 4*R*, 5*R*, 6*S*, 7*R*, 10*S*, and 7′*S* based on the ECD calculation (Figure S1).

**Figure 5:**
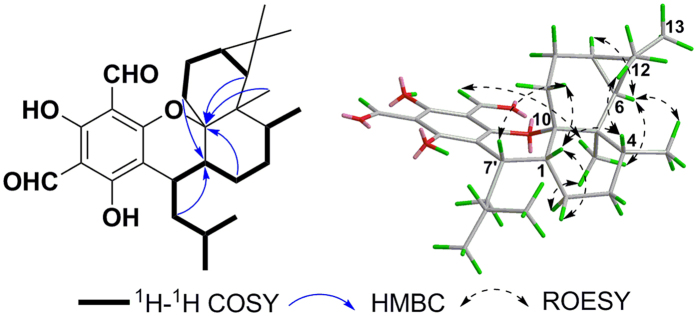
Key ^1^H-^1^H COSY, HMBC, and ROESY correlations of 4.

**Figure 6:**
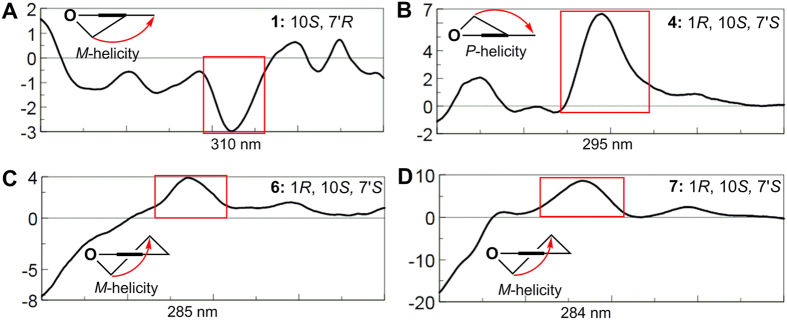
The *P/M*-helicity rules and ECD spectra of 1 (**A**), 4 (**B**), 6 (**C**), and 7 (**D**).

Careful analysis of NMR data (Tables 1 and 2) revealed that eucalrobusone M (**4**) had the very similar structure to **3**. However, the oxidized C-10 (*δ*_C_ 87.5) and 2D NMR correlations (Fig. 5) confirmed that the oxo bridge was between C-10 and C-3′ in **4**, led to an unusual 6/6/6/6/3 ring system. Another difference was the *β*-orientation of H-7′, which was assigned by the key ROESY correlations of H-7′/H-9*β* and H-1/H-9*β*. The other relative configurations of the chiral centers were determined the same as those of **3**, due to the ROESY correlations (Fig. 5). According to the Snatzke’s helicity rule, the absolute configurations of C-1, C-10, and C-7′ in **4** were assigned as 1*R*, 10*S*, and 7′*S* by ECD spectrum, in which positive Cotton effect at 295 nm (Δ*ε* = + 6.67, Fig. 6B) was observed^10^.

Eucalrobusone N (**5**) had the molecular formula C_28_H_38_O_5_ that was determined by HRESIMS analysis ([M-H]^−^, *m/z* 453.2645). Its NMR data (Tables 1 and 2) showed similarity to those of eucarobustol A^12^. The only difference between them was the location of double bond which was assigned between C-1 and C-10 in **5** by the HMBC correlations from H-7′ to C-2/C-10, from H_2_-8′ to C-1, and from H-2/H-6/H_2_-8/Me-14 to C-10. The ROESY correlations of H-6/Me-14, H-6/Me-13, H-6/H-7, and H-4/Me-12 established the same configuration of aristolane moiety to those of **3** and **4**. Obviously, **5** was the precursor of **3** and **4**, which therefore assigned the same absolute configurations of aristolane moiety. Meanwhile, the shielding effect of the benzene ring resulted in the diagnostically upfield shifted Me-14 (*δ*_H_ 1.02), which occurred under the condition of 7′*S* configuration (Figure S2)^4^.

The NMR data (Tables 1 and 2) analysis revealed that eucalrobusones O-P (**6–7**) were two FPMs of the same carbon skeleton with euglobal-III^19^, possessing germacrane-type moieties. Yet the Me-15 existed as terminal double bond in both **6** and **7** due to the HMBC correlations from H_2_-3 to C-15 and from H_2_-2/H-6 to C-4. Furthermore, the hydroxylation of C-5 (*δ*_C_ 72.6) in **6** was revealed by the HMBC correlations from H-5 to C-3/C-7/C-15, while the carbonylation of C-5 (*δ*_C_ 203.7) in **7** was established by the HMBC correlations from H_2_-3/H-7/H_2_-15 to C-5. Both of their relative configurations were consistent with euglobal-III, and the H-5 in **6** was elucidated as *β*-orientation by the key ROESY correlations of H-5/Me-12, H-7/Me-13, and H-5/H-15a. The absolute configurations of C-1, C-10, and C-7′ in **6** and **7** were assigned as 1*R*, 10*S*, and 7′*S* by the application of the Snatzke’s helicity rule (Fig. 6C,D).

Three known FPMs, macrocarpal C^20^, eucalyptal D^17^, and euglobal V^21^, were also isolated from this plant. Their structures were confirmed by comparison of their spectroscopic data with values reported in literature.

**Figure 7:**
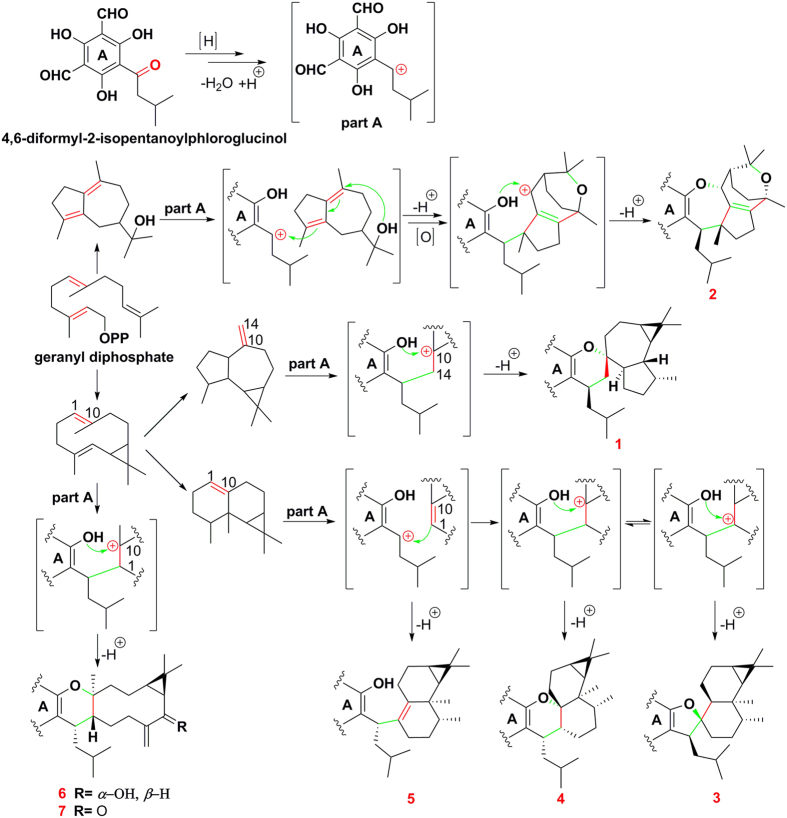
Plausible biosynthetic pathways of 1–7.

Plausible biosynthetic pathways of **1**–**7** were proposed (Fig. 7). The formation of carbocation in phloroglucinol precursor was thought to play a constructive role to trigger nucleophilic addition in the biosynthesis^4^,^11^,^17^. Thereafter, the different ion migration in terpene units made the cyclization diversify in oxa-heterocyclic rings. Although the dihydropyran rings of **1**, **6**, and **7** also could be deduced by the hetero Diels-Alder reaction^6^,^7^,^8^,^22^, but nucleophilic addition should be more responsible for those different coupling patterns and the diversified terpene moieties.

### Antifungal assays

Considering the reported antifungal effect of the genus *Eucalyptus*^2^,^9^,^23^,^24^, we tested the antifungal activities of all the isolates against *C. albicans* and *C. glabrata*. As results shown in Table 3, compounds **1**, **6**, and **8** exhibited significant antifungal activities against *C. glabrata*, and compounds **6** and **8** showed moderate antifungal activities against *C. albicans*. Obviously, the FPMs were important components responsible for the antifungal activities in the genus *Eucalyptus*, which were consistent with the literature^2^,^23^. In view of the moderate active **4**, as well as the inactive **3** and **5**, the coupling pattern of phloroglucinol and terpene might be important for their antifungal activities against *C. glabrata*. Observation of the structures and activities of compounds **6**–**7** indicated that the hydroxyl of C-5 should be a beneficial group for antifungal activity of **6**.

In conclusion, seven new FPMs (**1**–**7**) were obtained, of which **1**–**4** are noteworthy skeletons because of the unusual coupling patterns that formed diverse oxa-heterocyclic systems. Nucleophilic addition played an important role in their biosynthesis, which enabled the formation of different terpene carbocation that further lead to the structural variety. These FPMs showed different antifungal activities, which along with other reported ones, enlightened us to consider FPMs as antifungal precursors. But further researches, such as the structure-activity relationship, the *in vivo* evaluation, and the mechanism research should be carried out.

## Methods

### General experimental procedures

All optical rotation values were measured on a Jasco P-1020 polarimeter (Jasco, Tokyo, Japan). UV spectra were determined on a Shimadzu UV-2450 spectrophotometer (Shimadzu, Tokyo, Japan). ECD spectra were measured on a Jasco J-810 spectropolarimeter (Jasco, Tokyo, Japan) using spectroscopic grade solvent (MeOH). IR spectra (KBr pellets) were recorded on a Bruker Tensor 27 spectrometer (Bruker, Bremen, Germany). NMR spectra were obtained with a Bruker AVIII-500 spectrometer (Bruker, Bremen, Germany). CDCl_3_ and CD_3_OD with TMS as internal standard were used as solvent, and the signals of the residual solvent protons and the solvent carbon signals were used as internal references (*δ*_H_ = 7.26 and *δ*_C_ = 77.26 for CDCl_3_ and *δ*_H_ = 3.31 and *δ*_C_ = 49.00 for CD_3_OD, respectively). ESIMS and HRESIMS spectra were acquired on an Agilent 1100 series LC-MSD-Trap-SL mass analyzer and an Agilent UPLC-Q-TOF (Agilent Technologies, Palo Alto, CA, USA), respectively. TLC analyses were carried out using precoated silica gel GF_254_ plates (Qingdao Marine Chemical Plant, Qingdao, P. R. China). Column chromatographic methods were performed on Silica gel (200–300 mesh, Qingdao Haiyang Chemical Co., Ltd.), Sephadex LH-20 (Pharmacia, Sweden), and RP-C_18_ silica (40–63 *μ*m, FuJi, Japan). Preparative HPLC was performed on the LC-6A instrument that used a SPD-10A detector and a shim-pack RP-C_18_ column (10 *μ*m, 20 × 200 mm, Shimadzu, Tokyo, Japan). Analytical HPLC was performed on an Agilent 1200 Series instrument with a DAD detector by using a shim-pack VP-ODS column (250 × 4.6 mm, Agilent Technologies, Palo Alto, CA, USA).

### Plant material

The leaves of *E. robusta* were collected from Bozhou, Anhui Province, People’s Republic of China, in October 2013, which were authenticated by Prof. Mian Zhang of the Department of Medicinal Plants, China Pharmaceutical University. A voucher specimen (accession number DYA201310) was stored at the Department of Natural Medicinal Chemistry, China Pharmaceutical University.

### Extraction and isolation

The air-dried and powdered leaves of *E. robusta* (15.0 kg) were percolated with 95% EtOH for four times at room temperature. The combined EtOH residue (1.55 kg) was suspended in water and then extracted with petroleum ether (PE, 4.0 L × 4) and EtOAc (4.0 L × 4). The PE soluble partition (350 g) was fractionated by column chromatography over silica gel with a gradient of PE/EtOAc (100:1 to 1:2, *v/v*) to afford fractions A-E. Fr.B (70.8 g) was subsequently separated by silica gel column with PE/EtOAc (100:0 to 1:1, *v/v*) as eluent to yield seven subfractions (Frs.B1–7). Then, Fr.B3 (20.3 g) was separated by silica gel column using PE/EtOAc (100:1 to 1:1, *v/v*) as eluent to yield five subfractions (Frs.B3a-e). Fr.B3c (6.3 g) was then chromatographed on a RP-C_18_ column (MeOH/H_2_O, 60:40/100:0, *v/v*) to obtain eight fractions (Frs.B3c1-8). Fr.B3c6 (100.8 mg) was separated by preparative HPLC, with 90% methanol in water, to yield compounds **8** (5.1 mg, 25.5 min), **5** (10.2 mg, 31.0 min), and **6** (6.8 mg, 40.5 min). Fr.B3c7 (400.6 mg) was chromatographed over a Sephadex LH-20 column, eluted with CH_2_Cl_2_/MeOH (1:1, *v/v*) to yield five further fractions (Frs.B3c7a-e). Fr.B3c7c (90.3 mg) was purified by preparative HPLC using the mobile phase MeOH/H_2_O (95:5, *v/v*) to yield compounds **9** (3.5 mg, 30.5 min) and **4** (3.8 mg, 42.3 min). Fr.B3b (4.5 g) was applied to a silica gel column (PE/EtOAc, 100:0 to 1:1, *v/v*), then separated over Sephadex LH-20 (CH_2_Cl_2_/MeOH, 1:1, *v/v*), and purified finally by preparative HPLC (MeCN/H_2_O, 90:10, *v/v*) to give, in turn, **10** (5.3 mg, 28.0 min), **3** (3.4 mg, 33.7 min), and **7** (5.8 mg, 45.1 min). Fr.B3a (3.6 g) was applied to a silica gel column (PE/EtOAc, 100:0 to 1:1, *v/v*), purified finally by preparative HPLC (MeCN/H_2_O, 95:10, *v/v*) to yield compounds **1** (9.4 mg, 38.7 min) and **2** (2.8 mg, 50.1 min).

**Eucalrobusone J (1):** White powder; 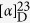 −45.1 (*c* 0.11, MeOH); UV (MeOH) *λ*_max_ (log *ε*) 205 (0.62), 278 (1.35) nm; ECD (MeOH, Δ*ε*) 200 (+1.58), 231 (−1.27), 268 (−1.42), 310 (−2.97) nm; IR (KBr) *ν*_max_ 3452, 2955, 1620, 1403 cm^−1^; ^1^H and ^13^C NMR data, see Tables 1 and 2; ESIMS *m/z* 453.2 [M-H]^−^; HRESIMS *m/z* 453.2648 ([M-H]^−^, C_28_H_37_O_5_; calcd 453.2646).

**Eucalrobusone K (2):** White powder; 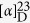 −2.0 (*c* 0.10, MeOH); UV (MeOH) *λ*_max_ (log *ε*) 205 (0.88), 269 (0.76) nm; ECD (MeOH, Δ*ε*) 207 (−6.08), 232 (+1.50), 282 (+2.86) nm; IR (KBr) *ν*_max_ 3450, 1720, 1235, 1015 cm^−1^; ^1^H and ^13^C NMR data, see Tables 1 and 2; ESIMS *m/z* 467.3 [M-H]^−^; HRESIMS *m/z* 467.2437 ([M-H]^−^, C_28_H_35_O_6_; calcd 467.2439).

**Eucalrobusone L (3):** White powder; 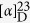 −2.4 (*c* 0.03, MeOH); UV (MeOH) *λ*_max_ (log *ε*) 192 (0.33), 205 (0.63), 278 (0.59), 382 (0.12) nm; ECD (MeOH, Δ*ε*) 214 (−0.99), 241 (+0.19), 273 (−0.39) nm; IR (KBr) *ν*_max_ 3445, 1640, 1400, 1050 cm^−1^; ^1^H and ^13^C NMR data, see Tables 1 and 2; ESIMS *m/z* 453.2 [M-H]^−^; HRESIMS *m/z* 453.2650 ([M-H]^−^, C_28_H_37_O_5_; calcd 453.2646).

**Eucalrobusone M (4):** White powder; 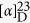 + 18.2 (*c* 0.03, MeOH); UV (MeOH) *λ*_max_ (log *ε*) 195 (0.12), 203 (0.23), 280 (0.12) nm; ECD (MeOH, Δ*ε*) 224 (+2.05), 295 (+6.67) nm; IR (KBr) *ν*_max_ 3440, 1635, 1405, 1055 cm^−1^; ^1^H and ^13^C NMR data, see Tables 1 and 2; ESIMS *m/z* 453.2 [M-H]^−^; HRESIMS *m/z* 453.2647 ([M-H]^−^, C_28_H_35_O_6_; calcd 453.2646).

**Eucalrobusone N (5):** White powder; 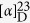 + 53.8 (*c* 0.13, MeOH); UV (MeOH) *λ*_max_ (log *ε*) 248 (0.42), 276 (1.29), 388 (0.39) nm; ECD (MeOH, Δ*ε*) 207 (+2.01), 224 (+7.99), 263 (−0.65), 300 (+0.33) nm; IR (KBr) *ν*_max_ 3443, 2924, 1632, 1400, 1057 cm^−1^; ^1^H and ^13^C NMR data, see Tables 1 and 2; ESIMS *m/z* 453.3 [M-H]^−^; HRESIMS *m/z* 453.2645 ([M-H]^−^, C_28_H_37_O_5_; calcd 453.2646).

**Eucalrobusone O (6):** White powder; 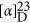 + 10.3 (*c* 0.07, MeOH); UV (MeOH) *λ*_max_ (log *ε*) 205 (0.58), 277 (0.96), 343 (0.11) nm; ECD (MeOH, Δ*ε*) 200 (−7.60), 285 (+3.96), 346 (+1.55) nm; IR (KBr) *ν*_max_ 3424, 2929, 1401 cm^−1^; ^1^H and ^13^C NMR data, see Tables 1 and 2; ESIMS *m/z* 469.4 [M-H]^−^; HRESIMS *m/z* 469.2599 ([M-H]^−^, C_28_H_37_O_6_; calcd 469.2596).

**Eucalrobusone P (7):** White powder; 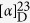 + 24.0 (*c* 0.10, MeOH); UV (MeOH) *λ*_max_ (log *ε*) 205 (0.72), 278 (1.02), 344 (0.14) nm; ECD (MeOH, Δ*ε*) 200 (−17.74), 284 (+8.64), 344 (+2.46) nm; IR (KBr) *ν*_max_ 3430, 2935, 1430 cm^−1^; ^1^H and ^13^C NMR data, see Tables 1 and 2; ESIMS *m/z* 467.3 [M-H]^−^; HRESIMS *m/z* 467.2441 ([M-H]^−^, C_28_H_35_O_6_; calcd 467.2439).

### Quantum chemical ECD calculation

The geometries generated based on NMR were optimized using MM2. The corresponding minimum geometries found were further re-optimized by DFT calculations at the B3LYP/6-31 G + (d, p) level. ECD computations were performed by means of the TD-SCF method under B3LYP/6-311 G + (d, 2p) level with 19 nm UV correction (*σ* = 0.39) for compound **2** and 22 nm UV correction (*σ* = 0.30) for compound **3**, respectively.

### Antifungal assays

The antifungal assay was performed by using broth microdilution method as previously reported^25^,^26^. Briefly, test compounds were serially diluted and co-incubated with standard microbial suspensions in 96-well plates for 24 h at 37 °C. The optical densities at 530 nm (OD530) were measured by using a spectrophotometer and growth inhibition of each concentration was calculated. The MIC_50_ value was defined as the minimum concentration of compound at which the growth of microorganism was half inhibited. All calculations were performed using GraphPad Prism 6 (GraphPad Software, San Diego, CA). Fluconazole and amphotericin B were used as the positive control in assays of all isolates antifungal activities against *C. albicans* and *C. glabrata*, respectively. All experiments were performed in three independent experiments.

## Additional Information

**How to cite this article**: Shang, Z.-C. *et al*. New Formyl Phloroglucinol Meroterpenoids from the Leaves of *Eucalyptus robusta. Sci. Rep.* **6**, 39815; doi: 10.1038/srep39815 (2016).

**Publisher's note:** Springer Nature remains neutral with regard to jurisdictional claims in published maps and institutional affiliations.

## Supplementary Material

Supporting Information

## Figures and Tables

**Figure 1:**
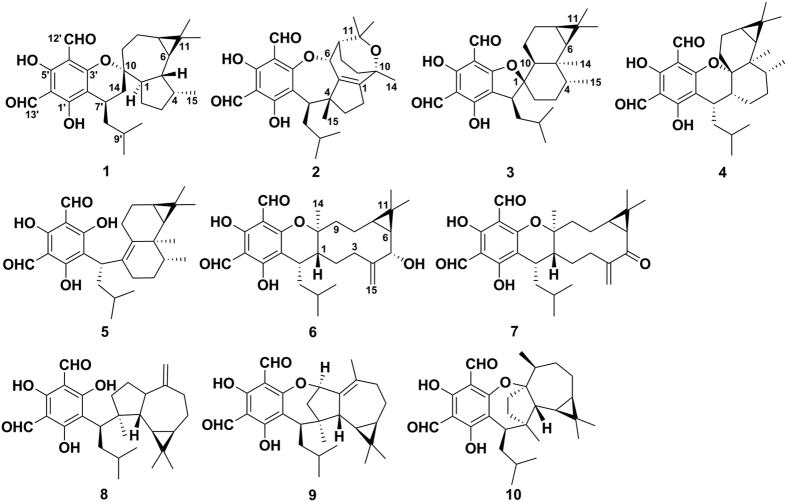
Structures of compounds 1–10.

**Table 1:** ^1^H NMR data for compounds 1–7 (500 MHz, *J* in Hz).

*no.*	1^a^	2^a^	3^a^	4^a^	5^b^	6^a^	7^a^
1	2.27, dd (17.2, 8.3)			2.27, m		2.05, m	1.92, m
2*α*	1.30, m	2.28^c^, m	1.98, m	1.63, m	2.22, m	1.88, m	2.00, m
2*β*	1.67, m	2.61, m	1.67, m	1.19^c^, m	2.13, m	1.64, m	1.34^c^, m
3*α*	1.27, m	2.11, m	1.66, m	1.35^c^, m (2 H)	1.32^c^, m (2 H)	2.60, m	2.82, br t (12.4)
3*β*	1.62, m	1.68, m	1.50, m			2.22, ddd (12.3, 9.1, 1.9)	2.23, m
4	2.02, m		1.58, m	1.77, m	1.70, m		
5	1.36^c^, m					3.86, d (10.6)	
6	0.61, br t	4.46, br s	0.40, d (9.6)	0.62, d (9.8)	0.59, d (9.2)	0.90, m	2.27, d (10.1)
7	0.70, m	2.29, m	0.73, td (9.6, 3.4)	0.95^c^, m	0.79, m	0.68, m	1.34^c^, m
8*α*	1.87, m	2.28^c^, m	2.03, m	1.92, m	1.99, m	1.75, m	1.61, m
8*β*	0.99^c^, m	1.31, m	1.34, m	1.13, m	1.40, m	1.63, m	1.03, m
9*α*	1.63^c^, m	1.88, m	1.17, m	1.47, m	1.86, m	2.09, m	2.06, m
9*β*	2.21, m	1.65, m	1.31, m	1.73, m	2.62, br dd (13.8, 3.3)	1.84, m	1.58, m
10			1.36, m				
12^d^	1.02, s	1.35, s	1.18, s	1.23, s	1.04, s	1.03, s	1.30, s
13	1.04, s	1.47, s	1.00, s	1.04, s	0.98, s	1.08, s	1.06, s
14*α*	1.80, m	1.28, s	1.15, s	1.19^c^, s	1.02, s	1.42, s	1.31, s
14*β*	2.05, m						
15a	0.93, d (7.1)	0.97, s	1.02, d (6.7)	0.95^c^, d (6.9)	0.96, d (6.9)	5.41, s	5.71, s
15b						5.08, s	5.50, s
7′	3.02, m	3.21, dd (11.2, 2.5)	3.29, dd (8.1, 4.7)	2.85, m	4.26, dd (10.1, 6.5)	3.02, m	3.05, m
8′a	1.63^c^, m	1.84, m	1.69, m	2.37, m	2.05, m	1.48, m	1.49, m
8′b	1.36^c^, m	1.41, m	1.52, m	1.23, m	1.25, m	1.38, m	1.34^c^, m
9′	1.71, m	1.15, m	1.70, m	1.67, m	1.38, m	1.80, m	1.85, m
10′	0.99^c^, d (6.3)	0.88, d (6.5)	0.96, d (5.5)	0.97, d (6.5)	0.86, d (6.5)	0.99, d (6.4)	0.97, d (6.4)
11′	0.94, d (6.3)	0.79, d (6.5)	0.95, d (5.5)	0.94, d (6.5)	0.84, d (6.5)	0.87, d (6.4)	0.88, d (6.4)
12′	10.03, s	10.11, s	9.84, s	10.01, s	9.89^c^, s	9.99, s	9.96, s
13′	10.14, s	10.27, s	10.10, s	10.12, s	9.89^c^, s	10.15, s	10.15, s
OH-1′	13.31, s	13.27, s	13.04, s	13.65, s		13.32, s	13.30, s
OH-5′	13.46, s	13.21, s	12.81, s	13.39, s		13.43, s	13.42, s

^a^Measured in CDCl_3_.

^b^Measured in CD_3_OD.

^c^Overlapped.

^d^*β*-oriented.

**Table 2:** ^13^C NMR data for compounds 1–7 (125 MHz).

*no.*	1^a^	2^a^	3^a^	4^a^	5^b^	6^a^	7^a^
1	45.3	148.3	98.9	31.5	133.9	39.3	29.0
2	26.5	32.2	33.3	20.7	28.2	31.8	38.6
3	34.8	34.5	27.4	25.3	28.5	34.7	31.0
4	36.9	51.8	40.2	34.3	38.8	154.6	154.3
5	39.1	137.4	38.7	42.2	38.9	72.6	203.7
6	28.5	85.8	34.8	36.0	35.6	32.7	34.8
7	26.9	43.0	19.7^c^	22.1	21.4	29.3	38.5
8	19.5	20.1	21.2	17.7	22.0	18.4	17.3
9	38.8	32.7	18.8	29.2	24.5	42.0	42.1
10	86.5	71.2	50.7	87.5	137.1	86.8	85.6
11	20.2	74.6	19.7^c^	20.5	19.3	19.1	27.3
12	16.1	32.0	16.5	17.5	17.2	15.5	15.6
13	28.8	31.0	31.7	31.9	30.5	29.4	29.6
14	23.1	28.4	18.7	21.5	23.8	25.6	25.1
15	16.0	25.6	16.1	16.4	17.0	114.2	120.2
1′	169.3	170.2	166.4	171.4	173.6	168.6	168.9
2′	106.0	117.8	109.1	104.3	106.4	108.9	108.9
3′	164.1	168.4	169.1	166.1	166.5	163.7	163.4
4′	104.5	108.3	101.1	104.7	108.8^c^	104.3	104.3
5′	168.5	167.6	169.5	168.3	167.7	168.5	168.5
6′	104.0	106.1	104.8	105.3	108.8^c^	103.9	104.1
7′	25.2	41.5	44.1	31.4	36.5	30.6	28.2
8′	42.9	39.3	38.4	35.9	42.1	42.1	42.2
9′	26.4	27.2	26.9	25.6	27.8	26.9	27.2
10′	21.8	22.1	22.5	21.6	24.0	21.7	21.9
11′	23.8	24.5	23.4	24.4	22.7	24.6	24.4
12′	192.5	193.5	190.8	192.6	192.4^c^	192.2	192.1
13′	191.9	192.8	191.8	191.9	192.4^c^	191.9	191.8

^a^Measured in CDCl_3_.

^b^Measured in CD_3_OD.

^c^Overlapped.

**Table 3:** Antifungal activities of compounds 1–10 against *C. albicans* and *C. glabrata*.

Compounds	*C. albicans*^a^	*C. glabrata*^a^
**1**	>50	2.57 ± 0.06
**2**	>50	>50
**3**	49.29 ± 4.78	>50
**4**	47.24 ± 5.60	15.40 ± 3.17
**5**	>50	>50
**6**	12.54 ± 2.14	1.95 ± 0.68
**7**	>50	30.56 ± 3.67
**8**	12.50 ± 2.25	2.49 ± 0.83
**9**	>50	>50
**10**	>50	>50
Fluconazole^b^	0.25 ± 0.12	
Amphotericin B^b^		0.26 ± 0.07

^a^MIC_50_ value of antifungal activities, *μ*g/mL.

^b^Positive control.
