# Supplementary material for: New Formyl Phloroglucinol Meroterpenoids from the Leaves of Eucalyptus robusta
Source: Sci Rep. 2016 Dec 22;6:39815. doi: 10.1038/srep39815 (PMC5177953; doi:10.1038/srep39815)

Supporting Information

New Formyl Phloroglucinol Meroterpenoids from the Leaves of *Eucalyptus robusta*

Zhi-Chun Shang[+], Ming-Hua Yang[+], Rui-Huan Liu, Xiao-Bing Wang, and Ling-Yi Kong*

State Key Laboratory of Natural Medicines, Department of Natural Medicinal Chemistry, China Pharmaceutical University, 24 Tong Jia Xiang, Nanjing 210009, People' s Republic of China.

*Corresponding author. Tel/Fax: +86-25-83271405. E-mail: cpu_lykong@126.com

[+]These authors contributed equally to this work.

Table of Contents

Figure S1 Calculated and experimental ECD spectra of **2** and **3**. **4**

Figure S2 The shielding effects of the benzene ring in *S*/*R*-7' of **5**. **4**

Table S1. Optimized Z-Matrixes of compound **2** in the Gas Phase (Å) at B3LYP/6-311G (d, 2p) level. **5**

Table S2. Optimized Z-Matrixes of compound **3** in the Gas Phase (Å) at B3LYP/6-311G (d, 2p) level. **6**

Figure S3-1 HRESIMS of eucalrobusone J (**1**). **9**

Figure S3-2 ^{1}H NMR spectrum of eucalrobusone J (**1**) in $CDCl_3$. **9**

Figure S3-3 ^{13}C NMR spectrum of eucalrobusone J (**1**) in $CDCl_3$. **10**

Figure S3-4 HSQC spectrum of eucalrobusone J (**1**) in $CDCl_3$. **10**

Figure S3-5 HMBC spectrum of eucalrobusone J (**1**) in $CDCl_3$. **11**

Figure S3-6 ^{1}H-^{1}H COSY spectrum of eucalrobusone J (**1**) in $CDCl_3$. **11**

Figure S3-7 ROESY spectrum of eucalrobusone J (**1**) in $CDCl_3$. **12**

Figure S4-1 HRESIMS of eucalrobusone K (**2**). **12**

Figure S4-2 ^{1}H NMR spectrum of eucalrobusone K (**2**) in $CDCl_3$. **13**

Figure S4-3 ^{13}C NMR spectrum of eucalrobusone K (**2**) in $CDCl_3$. **13**

Figure S4-4 HSQC spectrum of eucalrobusone K (**2**) in $CDCl_3$. **14**

Figure S4-5 HMBC spectrum of eucalrobusone K (**2**) in $CDCl_3$. **14**

Figure S4-6 ^{1}H-^{1}H COSY spectrum of eucalrobusone K (**2**) in $CDCl_3$. **15**

Figure S4-7 ROESY spectrum of eucalrobusone K (**2**) in $CDCl_3$. **15**

Figure S5-1 HRESIMS of eucalrobusone L (**3**). **16**

Figure S5-2 ^{1}H NMR spectrum of eucalrobusone L (**3**) in $CDCl_3$. **16**

Figure S5-3 ^{13}C NMR spectrum of eucalrobusone L (**3**) in $CDCl_3$. **17**

Figure S5-4 HSQC spectrum of eucalrobusone L (**3**) in $CDCl_3$. **17**

Figure S5-5 HMBC spectrum of eucalrobusone L (**3**) in $CDCl_3$. **18**

Figure S5-6 ^{1}H-^{1}H COSY spectrum of eucalrobusone L (**3**) in $CDCl_3$. **18**

Figure S5-7 ROESY spectrum of eucalrobusone L (**3**) in $CDCl_3$. **19**

Figure S6-1 HRESIMS of eucalrobusone M (**4**). **19**

Figure S6-2 ^{1}H NMR spectrum of eucalrobusone M (**4**) in $CDCl_3$. **20**

Figure S6-3 ^{13}C NMR spectrum of eucalrobusone M (**4**) in $CDCl_3$. **20**

Figure S6-4 HSQC spectrum of eucalrobusone M (**4**) in $CDCl_3$. **21**

Figure S6-5 HMBC spectrum of eucalrobusone M (**4**) in $CDCl_3$. **21**

Figure S6-6 ^{1}H-^{1}H COSY spectrum of eucalrobusone M (**4**) in $CDCl_3$. **22**

Figure S6-7 ROESY spectrum of eucalrobusone M (**4**) in $CDCl_3$. **22**

Figure S7-1 HRESIMS of eucalrobusone N (**5**). **23**

Figure S7-2 ^{1}H NMR spectrum of eucalrobusone N (**5**) in CD_3OD. **23**

Figure S7-3 ^{13}C NMR of eucalrobusone N (**5**) in CD_3OD. **24**

Figure S7-4 HSQC spectrum of eucalrobusone N (**5**) in CD_3OD. **24**

Figure S7-5 HMBC spectrum of eucalrobusone N (**5**) in CD_3OD. **25**

Figure S7-6 ROESY spectrum of eucalrobusone N (**5**) in CD_3OD. **25**

Figure S8-1 HRESIMS of eucalrobusone O (**6**). **26**

Figure S8-2 ^{1}H NMR spectrum of eucalrobusone O (**6**) in $CDCl_3$. **26**

Figure S8-3 ^{13}C NMR spectrum of eucalrobusone O (**6**) in $CDCl_3$. **27**

Figure S8-4 HSQC spectrum of eucalrobusone O (**6**) in $CDCl_3$. **27**

Figure S8-5 HMBC spectrum of eucalrobusone O (**6**) in $CDCl_3$. **28**

Figure S8-6 ROESY spectrum of eucalrobusone O (**6**) in $CDCl_3$. **28**

Figure S9-1 HRESIMS of eucalrobusone P (**7**). **29**

Figure S9-2 ^{1}H NMR spectrum of eucalrobusone P (**7**) in $CDCl_3$. **29**

Figure S9-3 ^{13}C NMR spectrum of eucalrobusone P (**7**) in $CDCl_3$. **30**

Figure S9-4 HSQC spectrum of eucalrobusone P (**7**) in $CDCl_3$. **30**

Figure S9-5 HMBC spectrum of eucalrobusone P (**7**) in $CDCl_3$. **31**

Figure S9-6 ROESY spectrum of eucalrobusone P (**7**) in $CDCl_3$. **31**

Figure S1 Calculated and experimental ECD spectra of **2** and **3**.

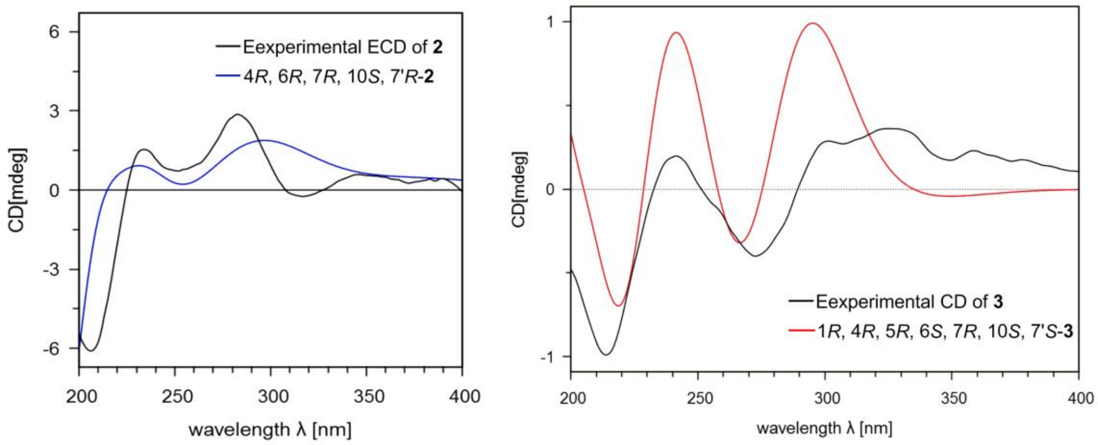

Figure S2 The shielding effects of the benzene ring in *S*/*R*-7' of **5**.

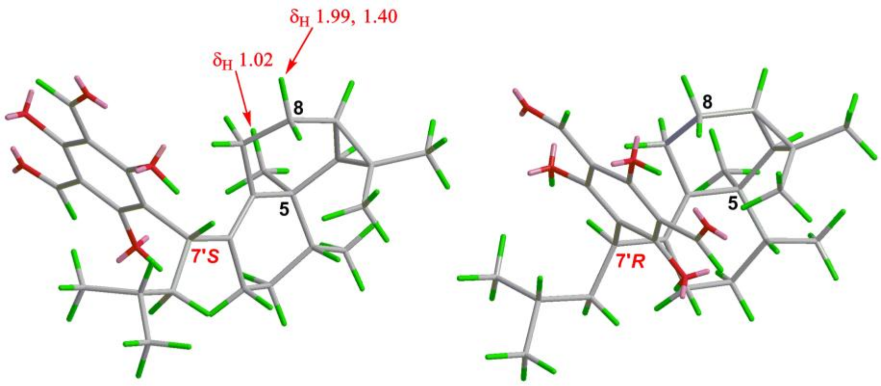

Table S1. Optimized Z-Matrixes of compound **2** in the Gas Phase (Å) at B3LYP/6-311G (d, 2p) level.

Center number	Atomic type	Coordinates (Angstroms)		
		X	Y	Z
1	C	-5.707524	0.612778	2.262583
2	C	-5.772108	-0.716155	2.024013
3	C	-4.732677	-1.381574	1.477269
4	C	-3.593286	-0.719181	1.185391
5	C	-3.557976	0.626806	1.305767
6	C	-4.592007	1.280681	1.889345
7	O	-6.771706	1.222045	2.866517
8	O	-4.837184	-2.707466	1.191245
9	C	-2.321944	-1.414305	0.668784
10	C	-1.985445	-2.836028	1.197726
11	C	-1.614898	-2.949048	2.697456
12	C	-2.751292	-2.613393	3.675513
13	C	-2.179505	-1.186204	-0.857710
14	C	-1.786381	0.246066	-1.201695
15	C	-2.366354	1.452790	-0.510266
16	O	-2.427474	1.295656	0.894879
17	C	-0.968002	-1.919618	-1.485780
18	C	-0.516348	-1.068711	-2.683543
19	C	-0.917368	0.306702	-2.223230
20	C	-3.461370	-1.567207	-1.620156
21	C	-0.430606	1.573127	-2.897524
22	C	-1.629167	2.440653	-3.312390
23	C	-2.107430	3.336784	-2.167996
24	C	-1.621045	2.761686	-0.828212
25	C	0.374685	1.251866	-4.168345
26	C	-0.078842	2.688839	-0.822356
27	O	0.433803	2.302255	-2.066222
28	C	0.511340	4.088135	-0.540757
29	C	0.501573	1.777039	0.272755
30	C	-1.099359	-4.370319	3.003032
31	C	-6.911223	-1.393761	2.354386
32	O	-7.112164	-2.577740	2.221449
33	C	-4.499398	2.631810	2.064520
34	O	-5.338501	3.359435	2.535951
35	H	6.613329	2.105055	3.217690
36	H	-4.169762	-2.931503	0.527389
37	H	-1.449335	-0.871205	1.116368
38	H	-1.074776	-3.200234	0.668954

39	H	-2.730684	-3.607445	0.923353
40	H	-0.772769	-2.240142	2.906213
41	H	-2.424923	-2.753361	4.731265
42	H	-3.072188	-1.552361	3.593014
43	H	-3.632701	-3.273754	3.516548
44	H	-3.415652	1.560989	-0.878471
45	H	-1.175703	-2.978151	-1.759619
46	H	-0.115043	-1.904786	-0.762827
47	H	0.580160	-1.175440	-2.840907
48	H	-1.061504	-1.333040	-3.617708
49	H	-4.327114	-0.931896	-1.329185
50	H	-3.340680	-1.450099	-2.720332
51	H	-3.731473	-2.630143	-1.428666
52	H	-1.372332	3.098541	-4.176187
53	H	-2.454070	1.779992	-3.670562
54	H	-3.215413	3.457882	-2.193551
55	H	-1.699726	4.365571	-2.303882
56	H	-1.933345	3.500943	-0.047400
57	H	0.722174	2.180570	-4.675846
58	H	-0.232096	0.680981	-4.907363
59	H	1.296631	0.670290	-3.943637
60	H	1.625052	4.072619	-0.557340
61	H	0.201106	4.472041	0.457508
62	H	0.202992	4.842274	-1.297525
63	H	1.613401	1.830897	0.298931
64	H	0.264570	0.702439	0.133498
65	H	0.132103	2.075494	1.279741
66	H	-0.784678	-4.470494	4.066969
67	H	-0.214636	-4.629809	2.377905
68	H	-1.889133	-5.131857	2.810137
69	H	-7.755709	-0.821277	2.795346
70	H	-3.572395	3.153649	1.746073

Table S2. Optimized Z-Matrixes of compound **3** in the Gas Phase (Å) at B3LYP/6-311G (d, 2p) level.

Center number	Atomic type	Coordinates (Angstroms)		
		X	Y	Z
1	C	0.328290	-0.700749	2.595669
2	C	-0.370297	-1.318051	1.376941
3	C	-0.148019	-0.505442	0.085697
4	C	1.367235	-0.426357	-0.256509
5	C	2.320225	-0.266283	0.987592
6	C	1.525108	0.200885	2.241666

7	C	1.645902	0.510393	-1.448446
8	C	3.135519	0.719098	-1.707185
9	C	4.019644	1.078810	-0.534080
10	C	3.590184	0.605991	0.832545
11	C	3.653728	2.092038	0.532211
12	C	4.843960	2.858317	1.110264
13	C	2.420257	2.969735	0.426711
14	C	2.897372	-1.691361	1.271462
15	C	2.360289	0.453961	3.513159
16	C	-3.910416	1.910904	-0.129644
17	C	-2.652707	1.914723	0.374480
18	C	-1.826228	0.915486	0.025232
19	C	-2.222247	-0.056087	-0.800038
20	C	-3.465247	-0.091147	-1.308969
21	C	-4.310067	0.911963	-0.962160
22	O	-4.757397	2.915702	0.246953
23	O	-0.656130	0.797086	0.382798
24	C	-1.064843	-0.991571	-1.072473
25	O	-3.767101	-1.106212	-2.16562
26	C	-1.334704	-2.502791	-1.154525
27	C	-1.359842	-3.086432	-2.584868
28	C	0.054925	-3.179682	-3.187091
29	C	-2.015035	-4.479033	-2.580980
30	C	-5.584388	0.934919	-1.455016
31	O	-6.113385	0.131056	-2.189114
32	C	-2.243415	2.899084	1.229746
33	O	-1.170298	2.998007	1.784970
34	H	0.654116	-1.520624	3.278915
35	H	-0.409139	-0.096388	3.178432
36	H	-1.459083	-1.429049	1.593595
37	H	0.006873	-2.355155	1.244845
38	H	1.587068	-1.437365	-0.688311
39	H	1.101105	1.201938	1.991402
40	H	1.230437	0.081706	-2.391053
41	H	1.114235	1.478495	-1.366545
42	H	3.275963	1.492638	-2.499931
43	H	3.538767	-0.239992	-2.118079
44	H	5.084623	1.029662	-0.800423
45	H	4.384634	0.277564	1.516951
46	H	4.606180	3.212603	2.138737
47	H	5.070660	3.742508	0.472376
48	H	5.768590	2.242970	1.178120
49	H	1.463319	2.435095	0.320305

50	H	2.305854	3.568623	1.358171
51	H	2.532004	3.676785	-0.425889
52	H	2.123546	-2.473090	1.413401
53	H	3.545123	-2.030970	0.430212
54	H	3.530818	-1.734279	2.183800
55	H	3.146755	1.225376	3.357615
56	H	1.715815	0.833608	4.339191
57	H	1.715815	0.833608	4.339191
58	H	-5.679487	2.778680	-0.008769
59	H	-0.675929	-0.645968	-2.055315
60	H	-4.604165	-1.025272	-2.636493
61	H	-0.521472	-3.066951	-0.645445
62	H	-2.270403	-2.737640	-0.597982
63	H	-1.967329	-2.430530	-3.253507
64	H	0.022601	-3.581820	-4.225371
65	H	0.553537	-2.186574	-3.238384
66	H	0.708668	-3.852458	-2.587184
67	H	-2.048117	-4.913749	-3.606019
68	H	-3.064183	-4.429632	-2.209492
69	H	-1.453732	-5.185770	-1.928484
70	H	-6.227146	1.791479	-1.153890
71	H	-2.984660	3.701481	1.450475

Figure S3-1 HRESIMS of eucalrobusone J (**1**).

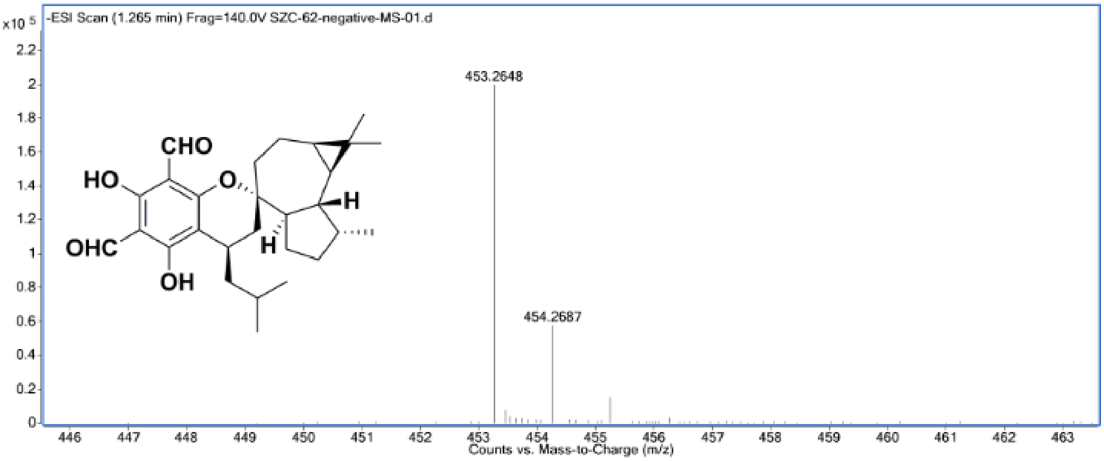

Elemental Composition Calculator

**Target m/z:**	453.2648	**Result type:**	Negative ions	**Species:**	[M-H]⁻
**Elements:**		C (0-80); H (0-120); O (0-30); N(0-10); Cl (0-5)			
**Ion Formula**		**Calcalated m/z**		**PPM Error**	
C28H37O5		453.2646		-0.24	

Figure S3-2 ^{1}H NMR spectrum of eucalrobusone J (**1**) in $CDCl_3$.

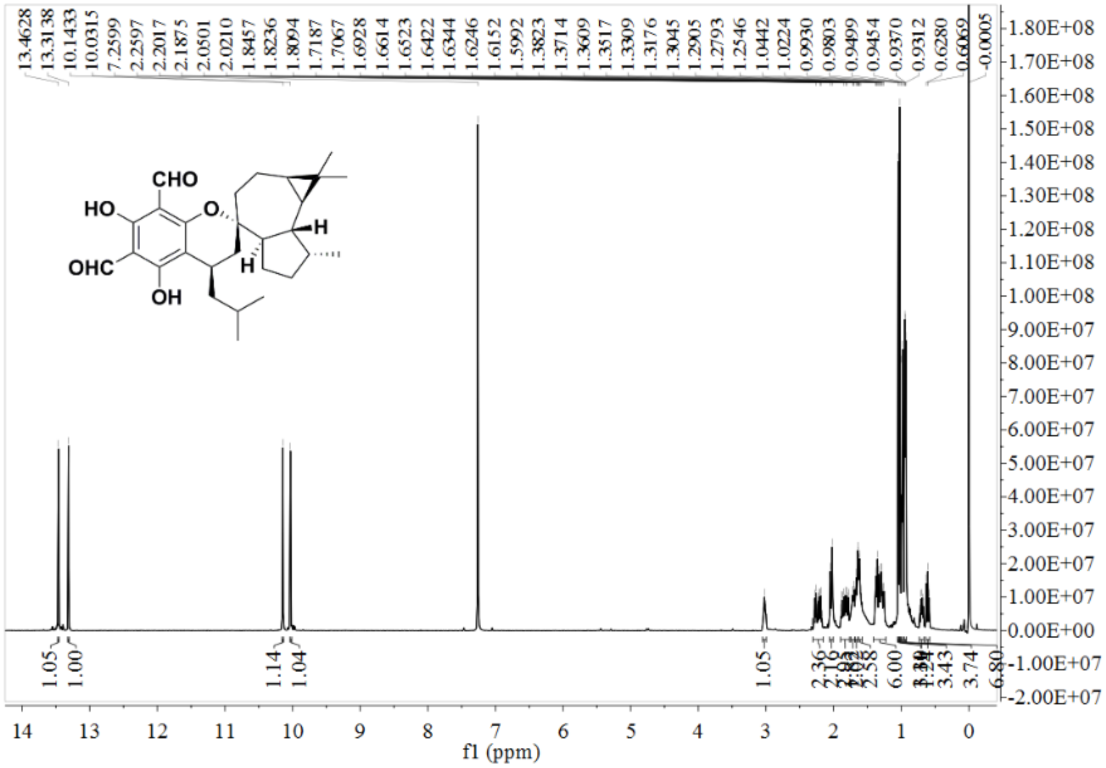

Figure S3-3 ^{13}C NMR spectrum of eucalrobusone J (**1**) in $CDCl_3$.

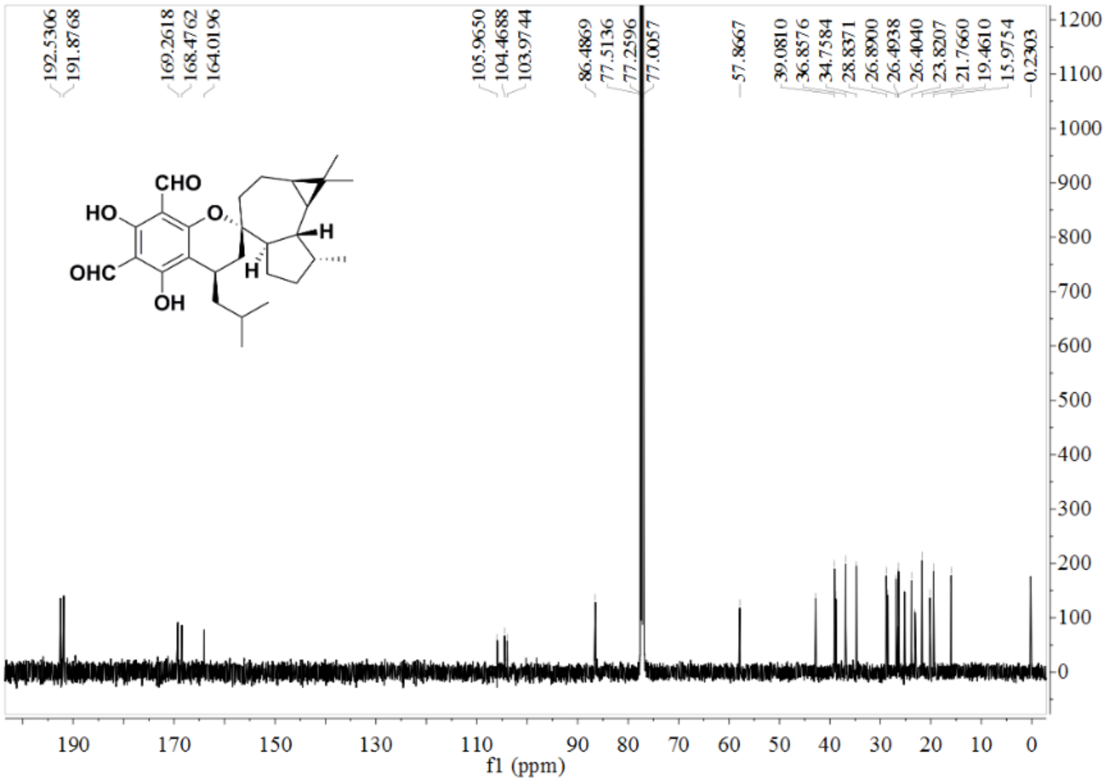

Figure S3-4 HSQC spectrum of eucalrobusone J (**1**) in $CDCl_3$.

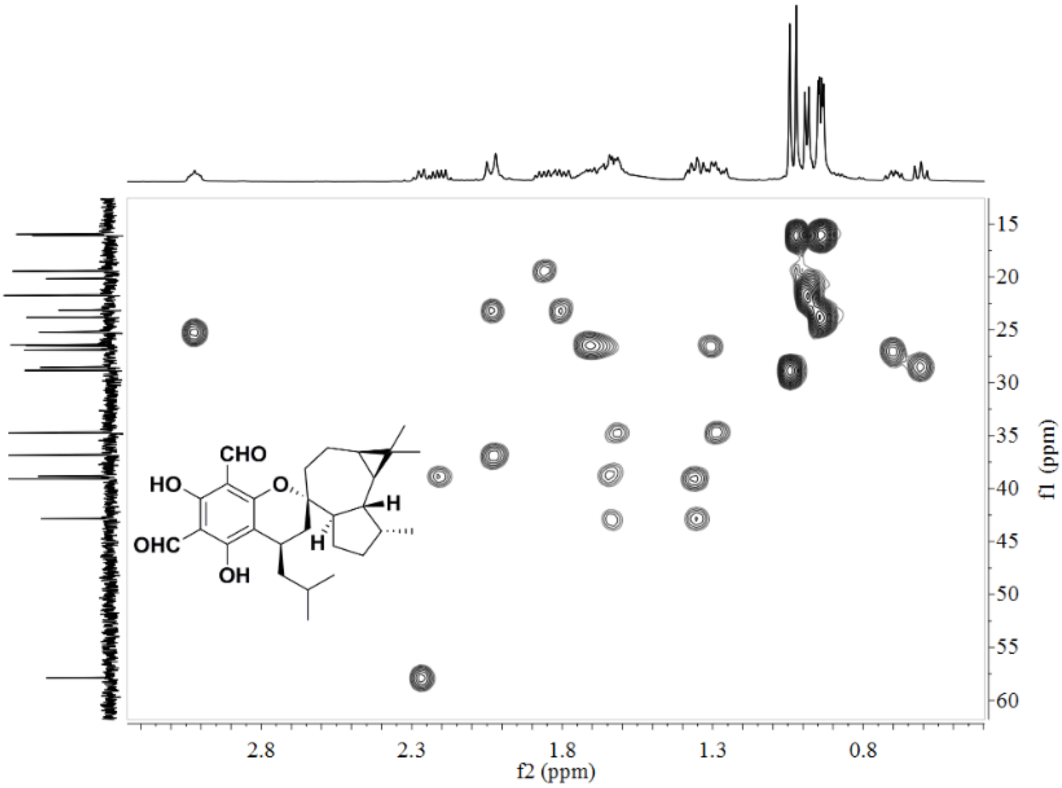

Figure S3-5 HMBC spectrum of eucalrobusone J (**1**) in $CDCl_3$.

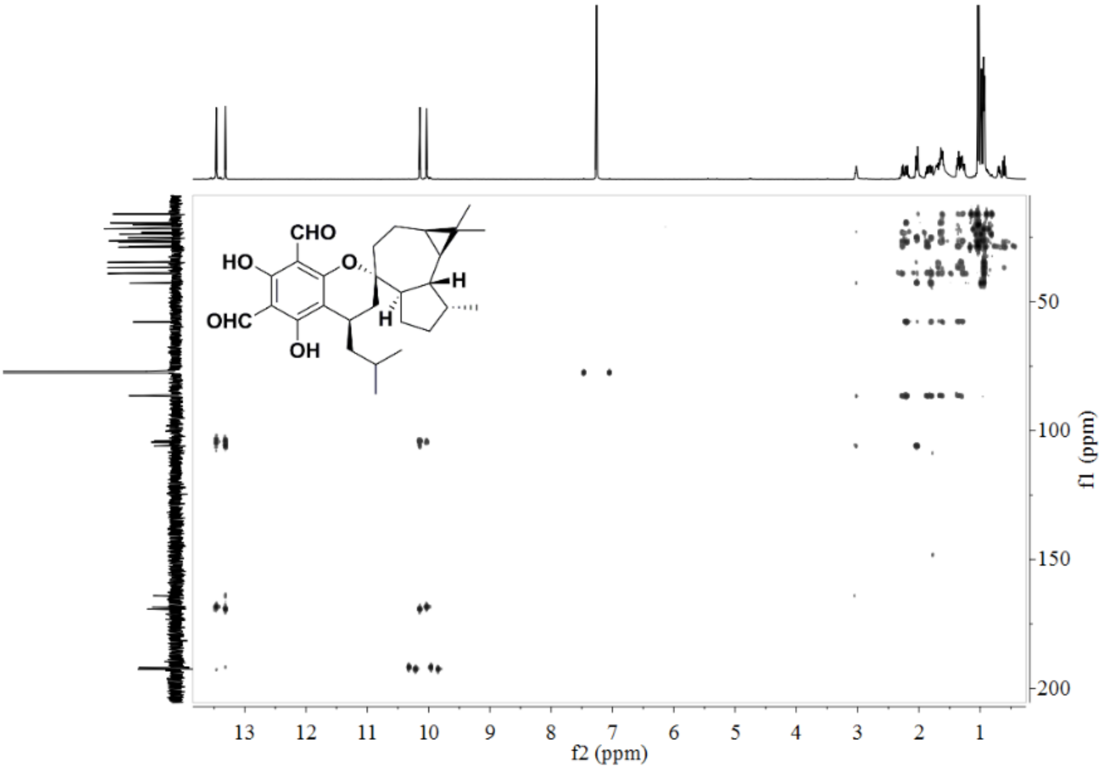

Figure S3-6 ^1H-^1H COSY spectrum of eucalrobusone J (**1**) in $CDCl_3$.

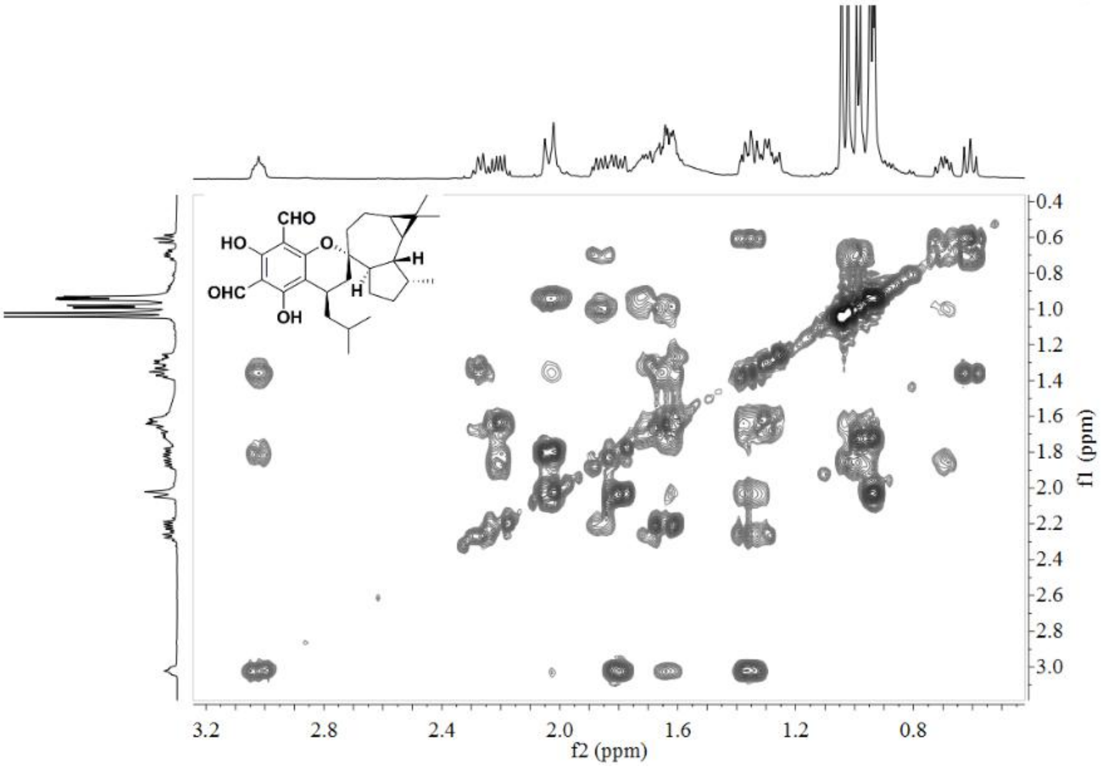

Figure S3-7 ROESY spectrum of eucalrobusone J (**1**) in $CDCl_3$.

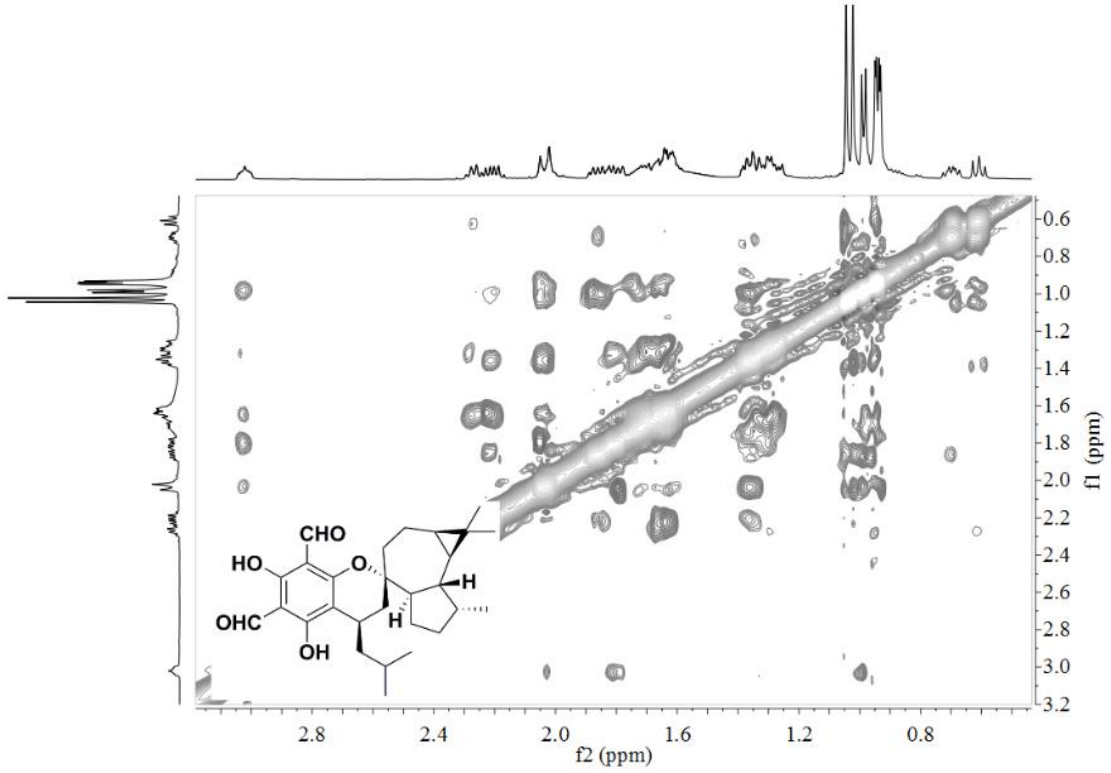

Figure S4-1 HRESIMS of eucalrobusone K (**2**).

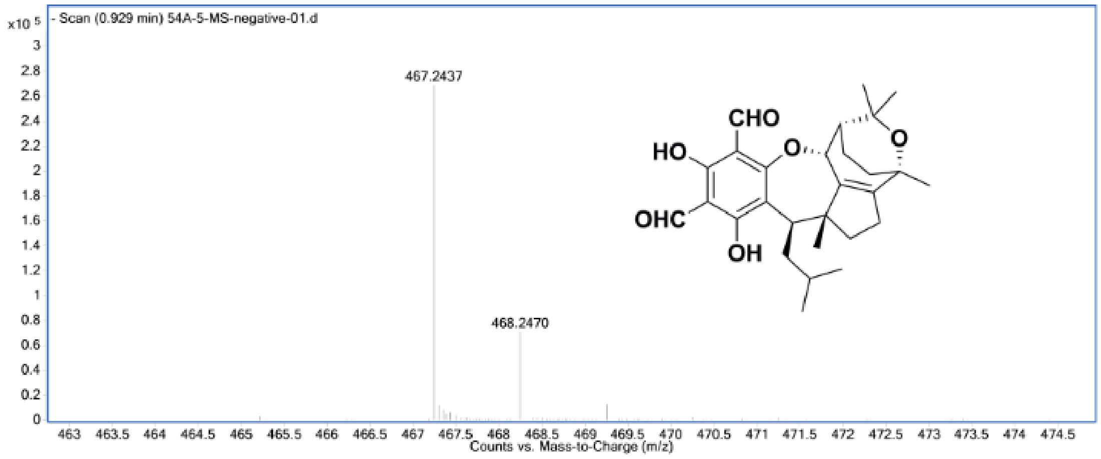

Elemental Composition Calculator

**Target m/z:**	467.2437	**Result type:**	Negative ions	**Species:**	[M-H]-
**Elements:**		C (0-80); H (0-120); O (0-30); N(0-10); Cl (0-5)			
**Ion Formula**		**Calcalated m/z**		**PPM Error**	
C28H35O6		467.2439		0.41	

Figure S4-2 ^{1}H NMR spectrum of eucalrobusone K (**2**) in $CDCl_3$.

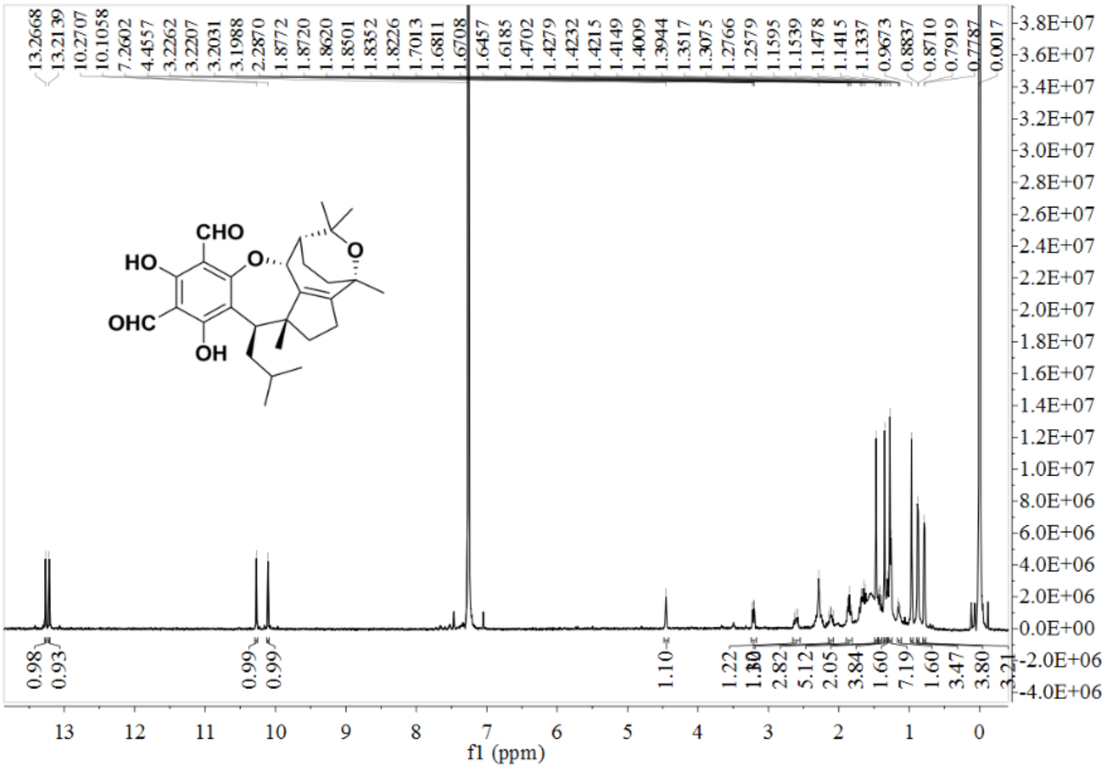

Figure S4-3 ^{13}C NMR spectrum of eucalrobusone K (**2**) in $CDCl_3$.

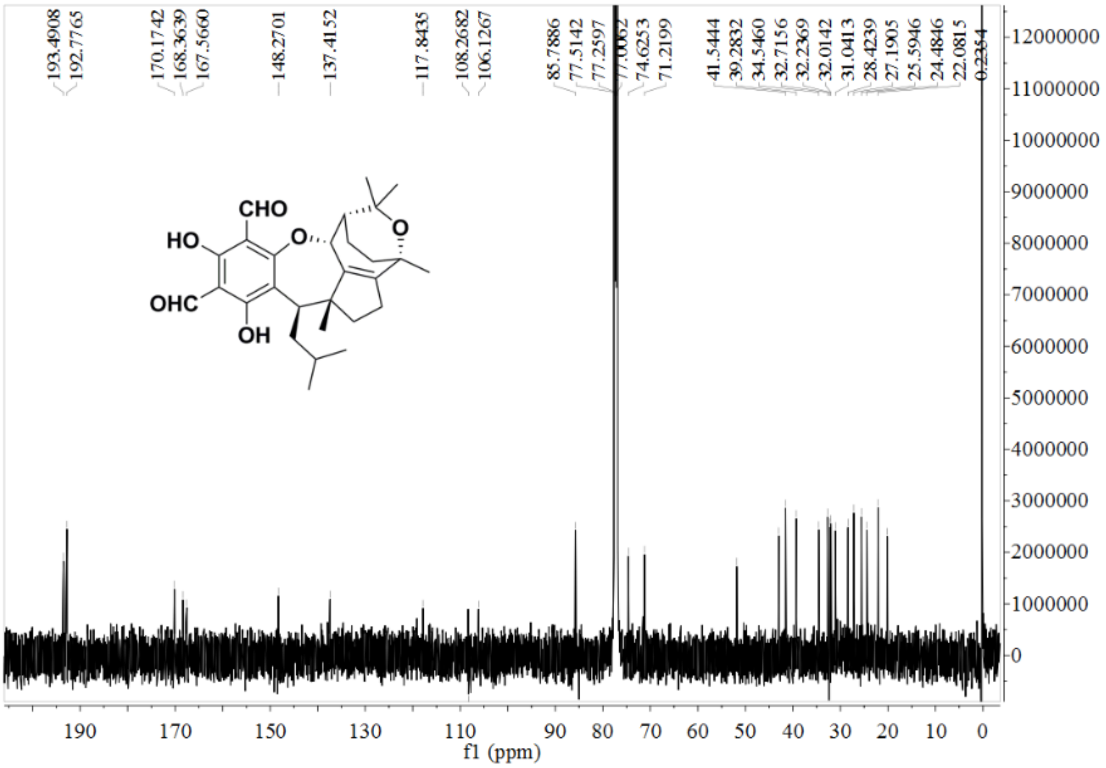

Figure S4-4 HSQC spectrum of eucalrobusone K (**2**) in $CDCl_3$.

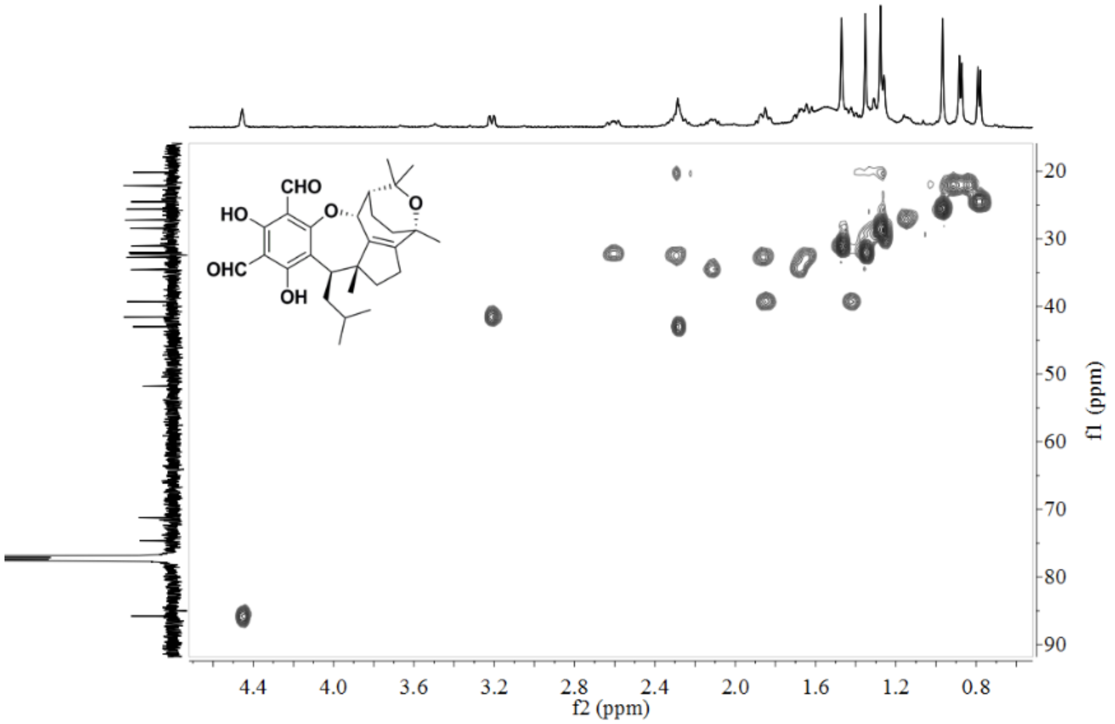

Figure S4-5 HMBC spectrum of eucalrobusone K (**2**) in $CDCl_3$.

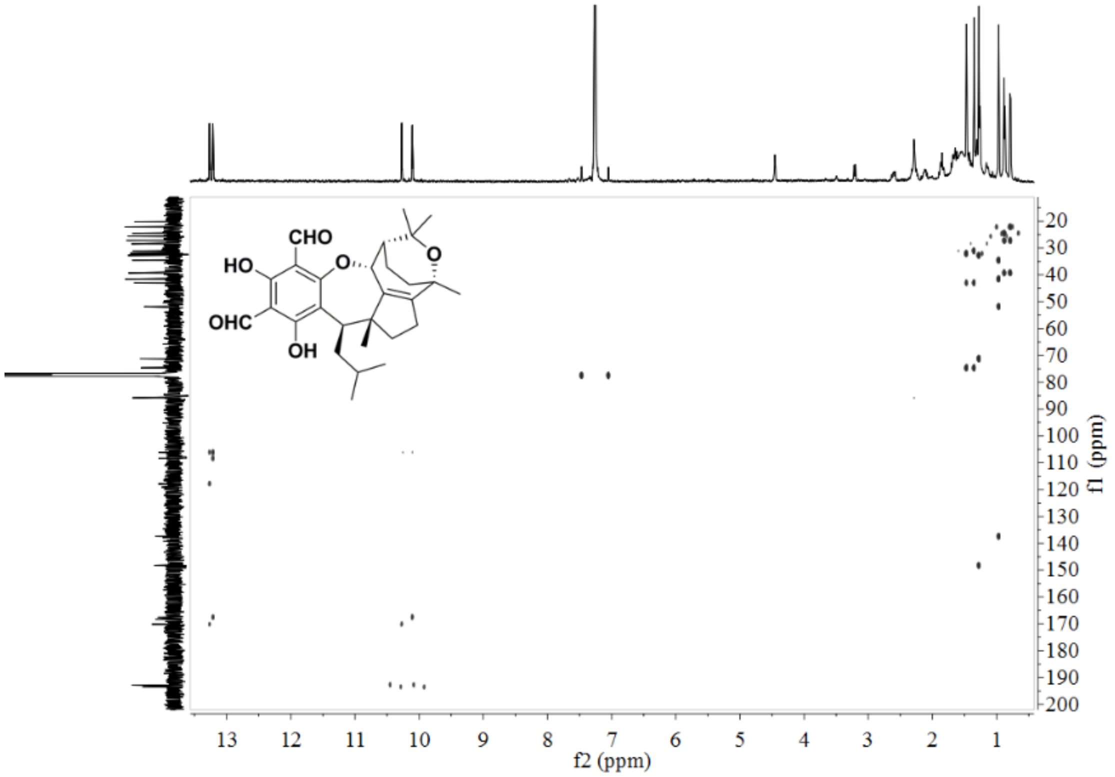

Figure S4-6 ^{1}H-^{1}H COSY spectrum of eucalrobusone K (**2**) in CDCl$_3$.

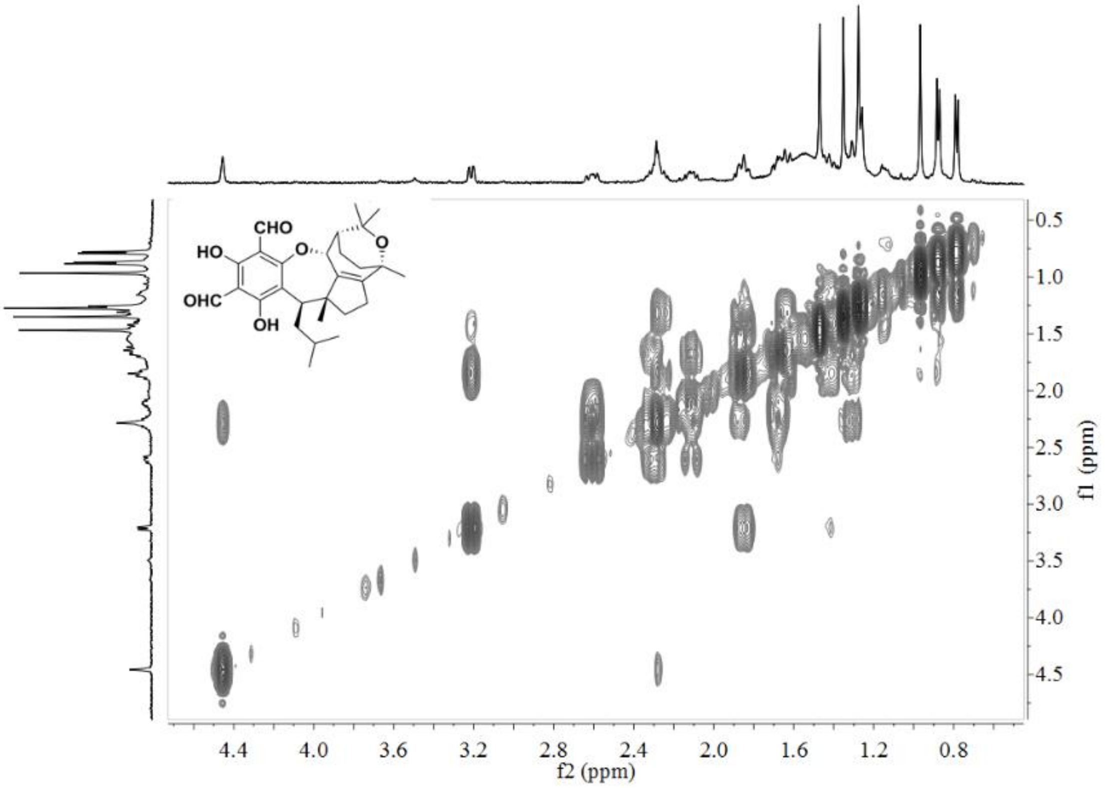

Figure S4-7 ROESY spectrum of eucalrobusone K (**2**) in CDCl$_3$.

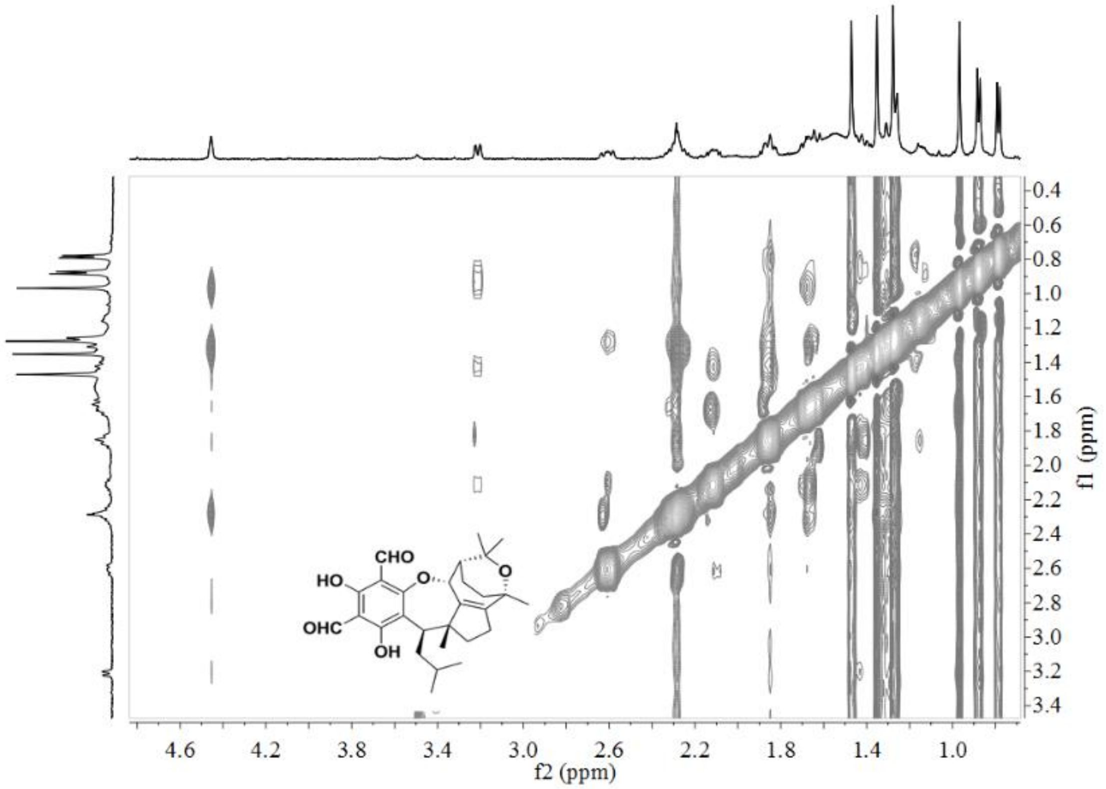

Figure S5-1 HRESIMS of eucalrobusone L (**3**).

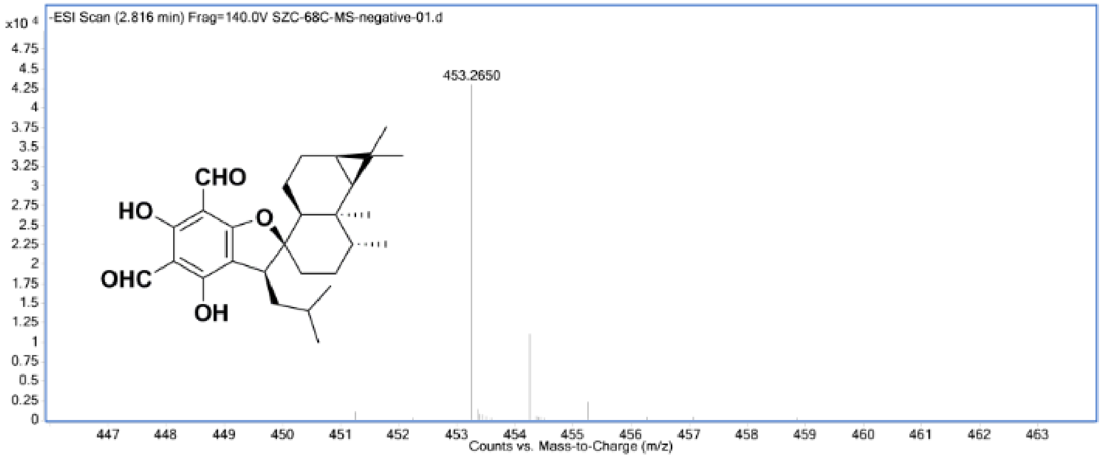

Elemental Composition Calculator

Target m/z:	453.2650	Result type:	Negative ions	Species:	[M-H]⁻
Elements:		C (0-80); H (0-120); O (0-30); N(0-10); Cl (0-5)			
Ion Formula		Calcalated m/z		PPM Error	
C28H37O5		453.2646		-0.73	

Figure S5-2 ^{1}H NMR spectrum of eucalrobusone L (**3**) in $CDCl_3$.

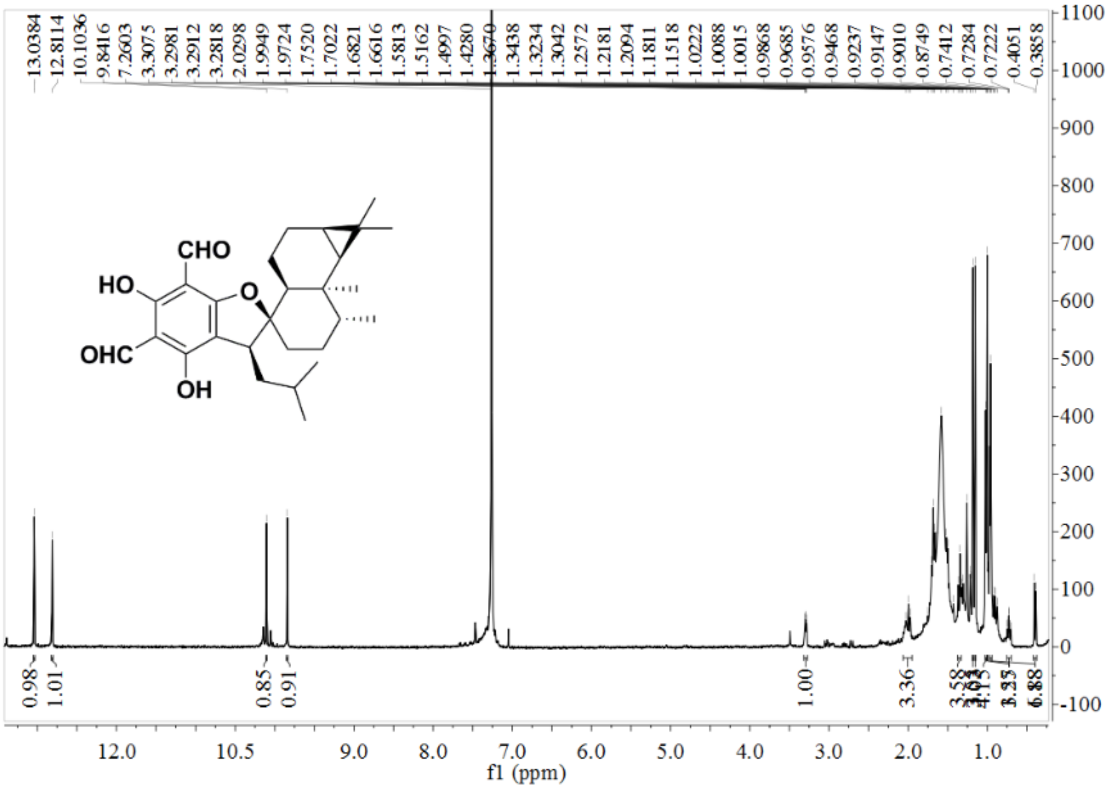

Figure S5-3 ^{13}C NMR spectrum of eucalrobusone L (**3**) in $CDCl_3$.

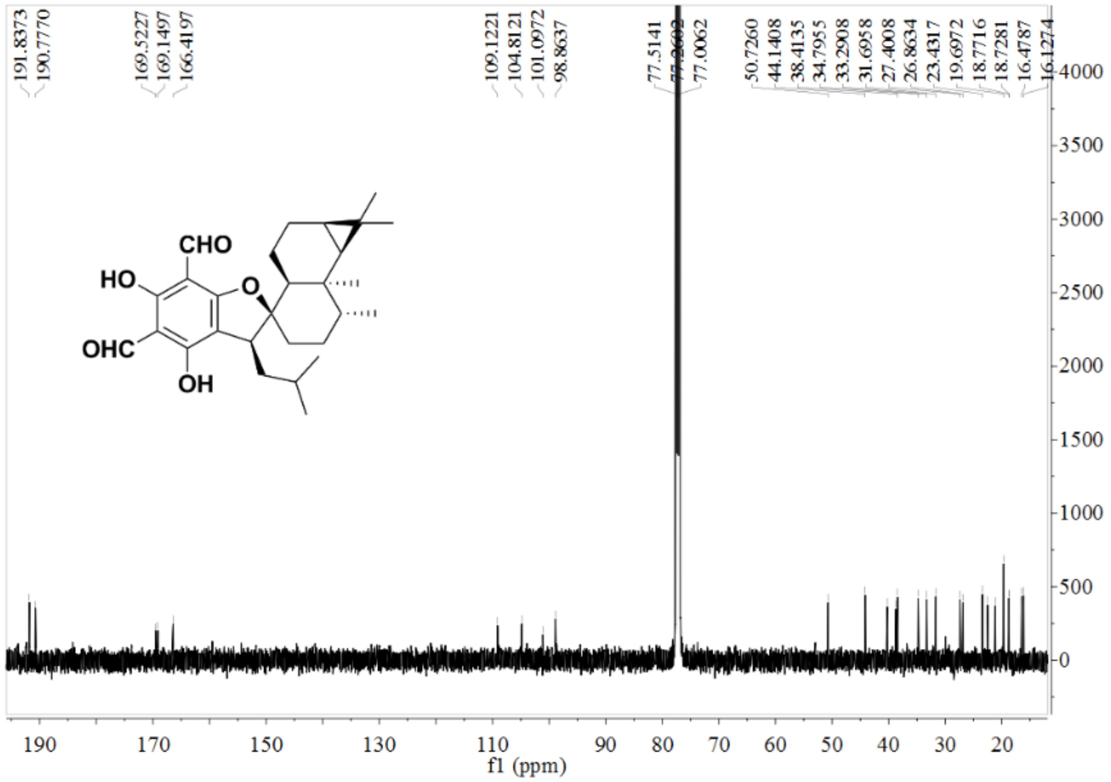

Figure S5-4 HSQC spectrum of eucalrobusone L (**3**) in $CDCl_3$.

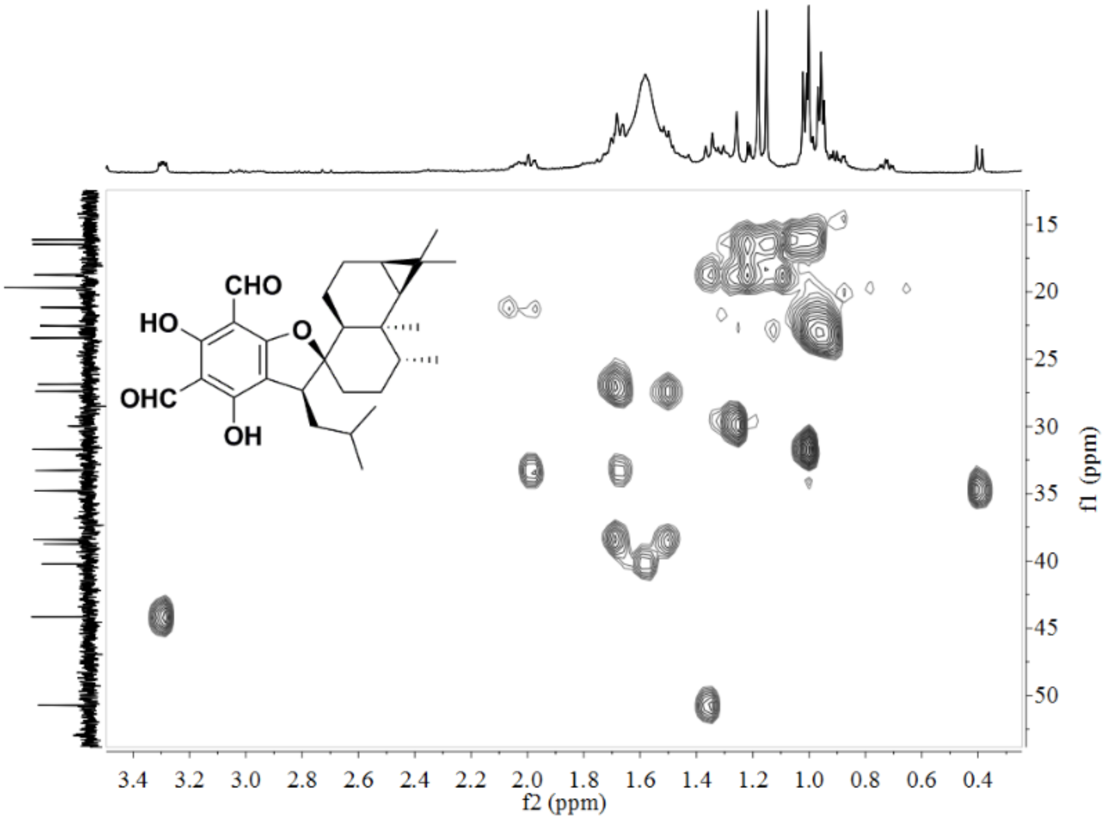

Figure S5-5 HMBC spectrum of eucalrobusone L (**3**) in $CDCl_3$.

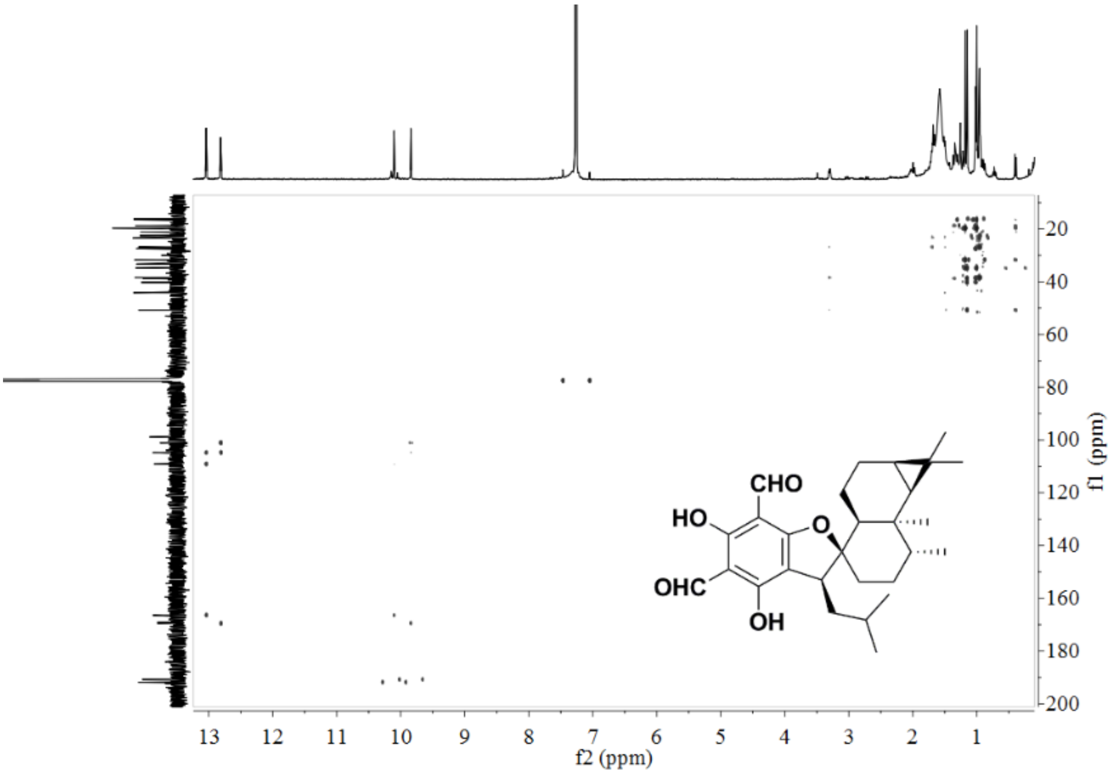

Figure S5-6 ^1H-^1H COSY spectrum of eucalrobusone L (**3**) in $CDCl_3$.

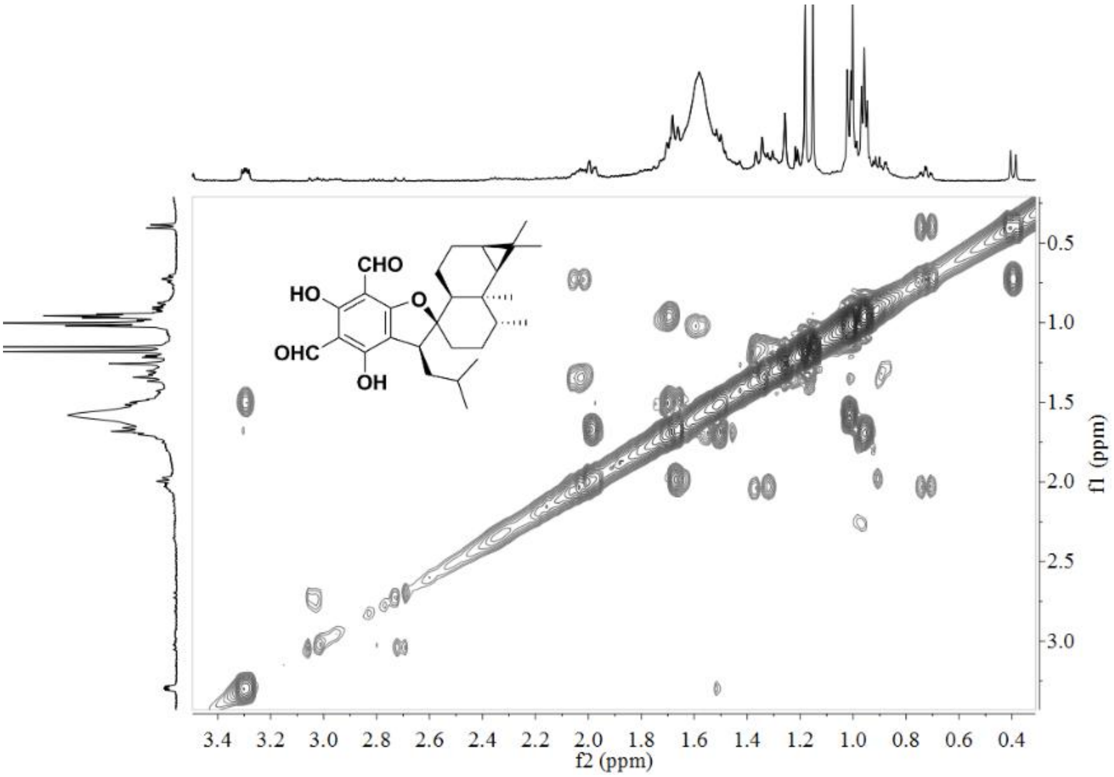

Figure S5-7 ROESY spectrum of eucalrobusone L (**3**) in $CDCl_3$.

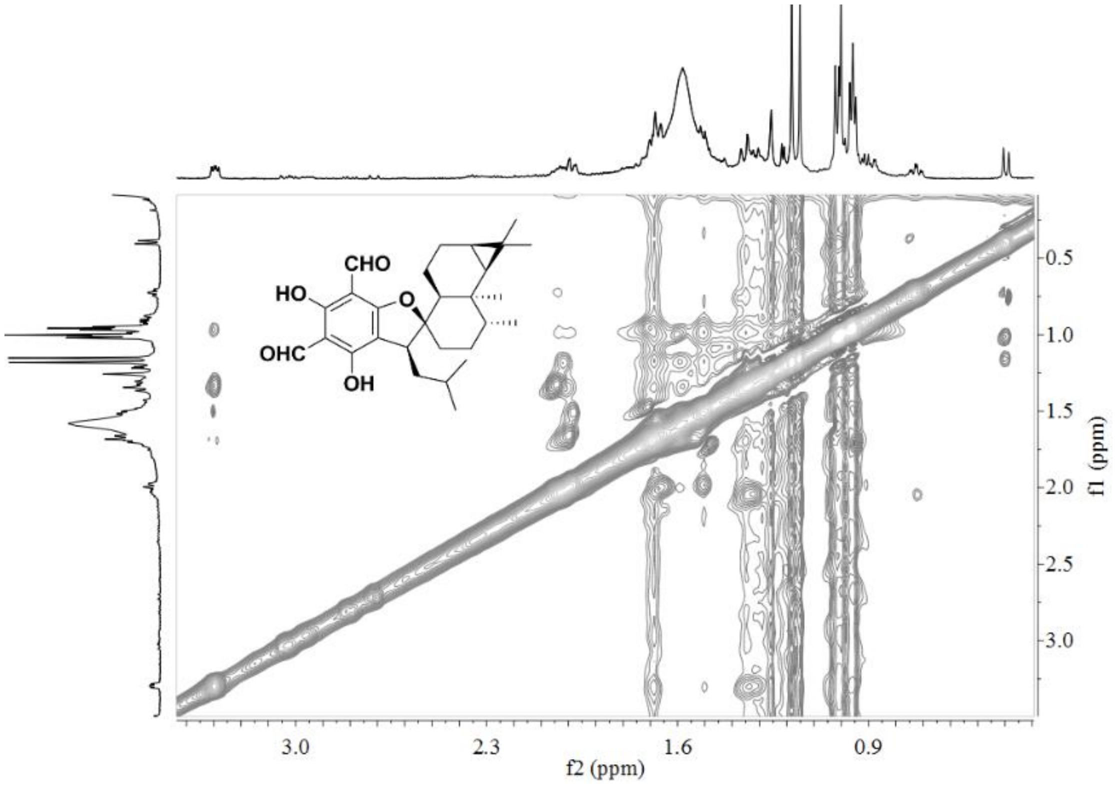

Figure S6-1 HRESIMS of eucalrobusone M (**4**).

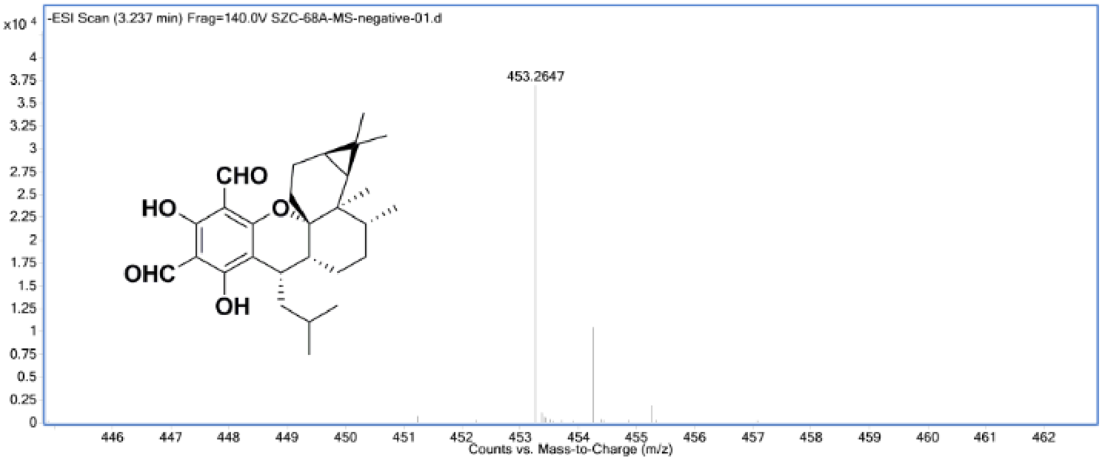

Elemental Composition Calculator

**Target m/z:**	453.2647	**Result type:**	Negative ions	**Species:**	$[M-H]^-$
**Elements:**		C (0-80); H (0-120); O (0-30); N(0-10); Cl (0-5)			
**Ion Formula**		**Calcalated m/z**		**PPM Error**	
C28H37O5		453.2646		-0.14	

Figure S6-2 ^{1}H NMR spectrum of eucalrobusone M (**4**) in $CDCl_3$.

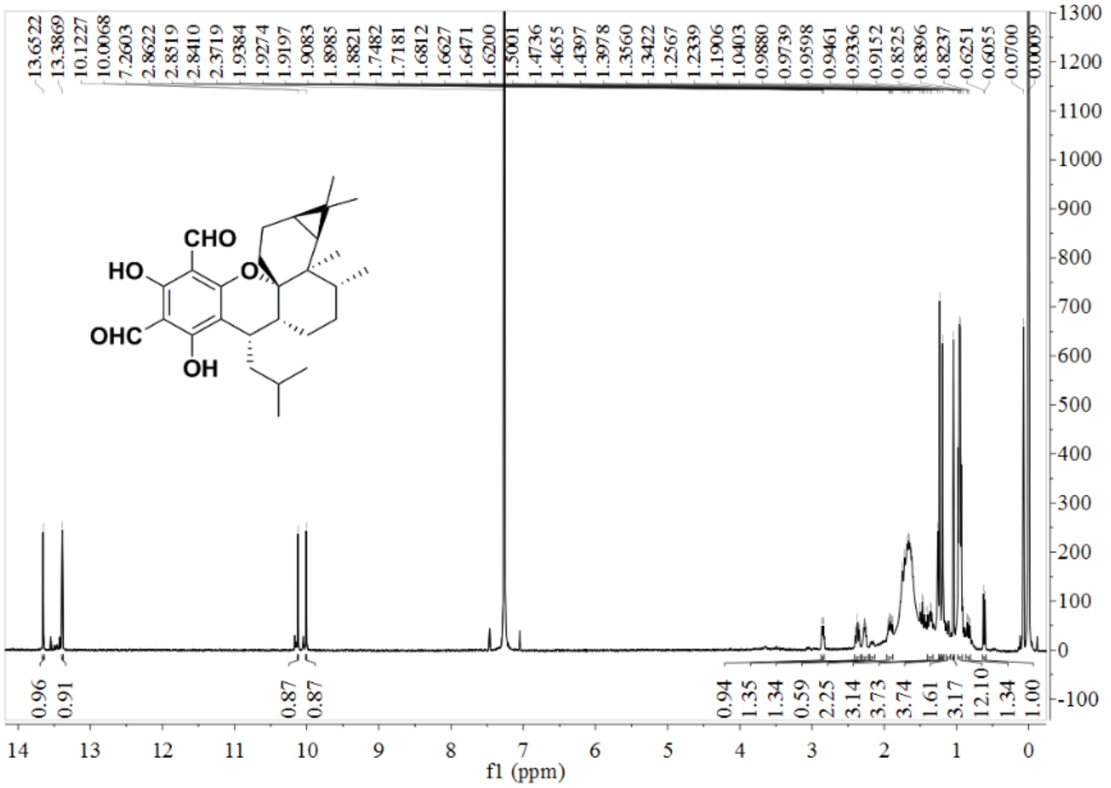

Figure S6-3 ^{13}C NMR spectrum of eucalrobusone M (**4**) in $CDCl_3$.

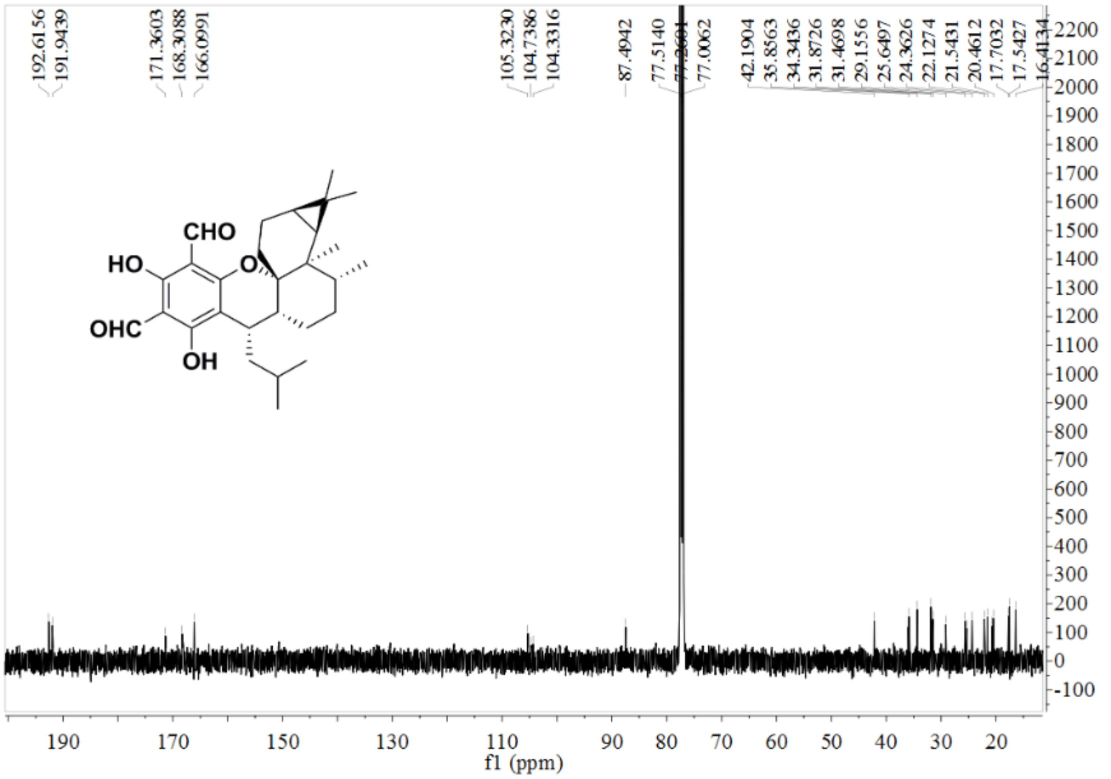

Figure S6-4 HSQC spectrum of eucalrobusone M (**4**) in $CDCl_3$.

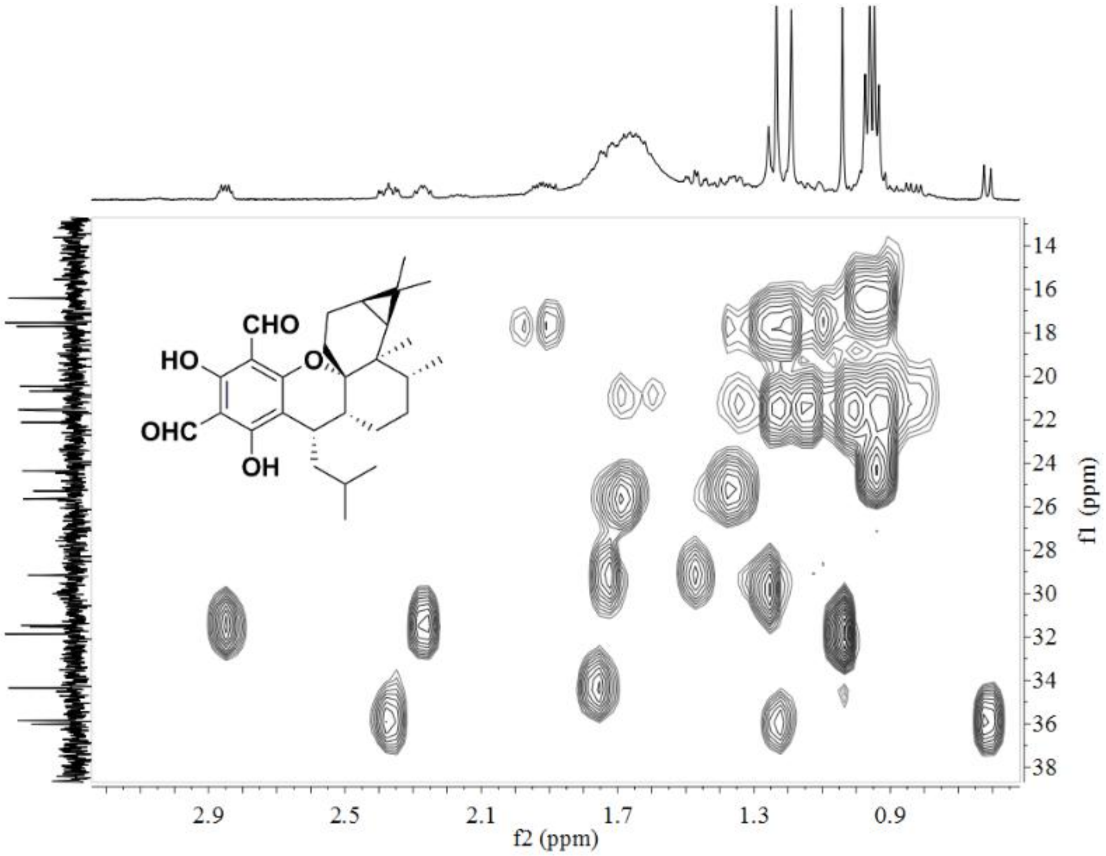

Figure S6-5 HMBC spectrum of eucalrobusone M (**4**) in $CDCl_3$.

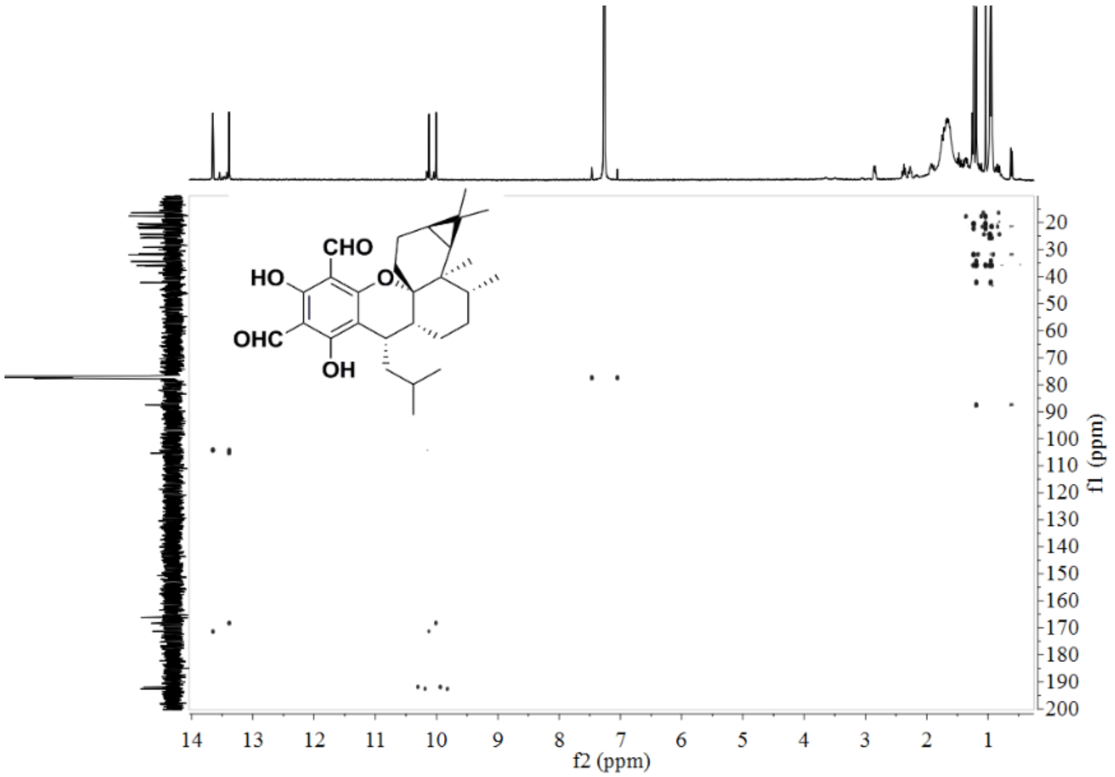

Figure S6-6 ^{1}H-^{1}H COSY spectrum of eucalrobusone M (**4**) in $CDCl_3$.

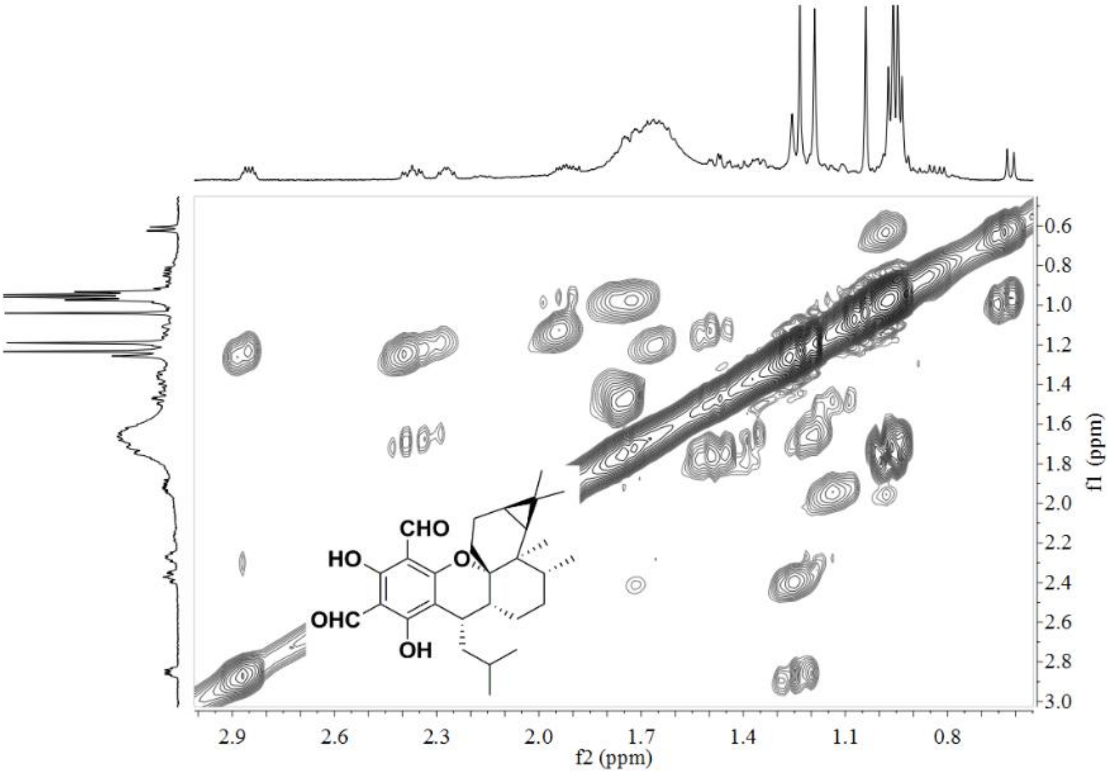

Figure S6-7 ROESY spectrum of eucalrobusone M (**4**) in $CDCl_3$.

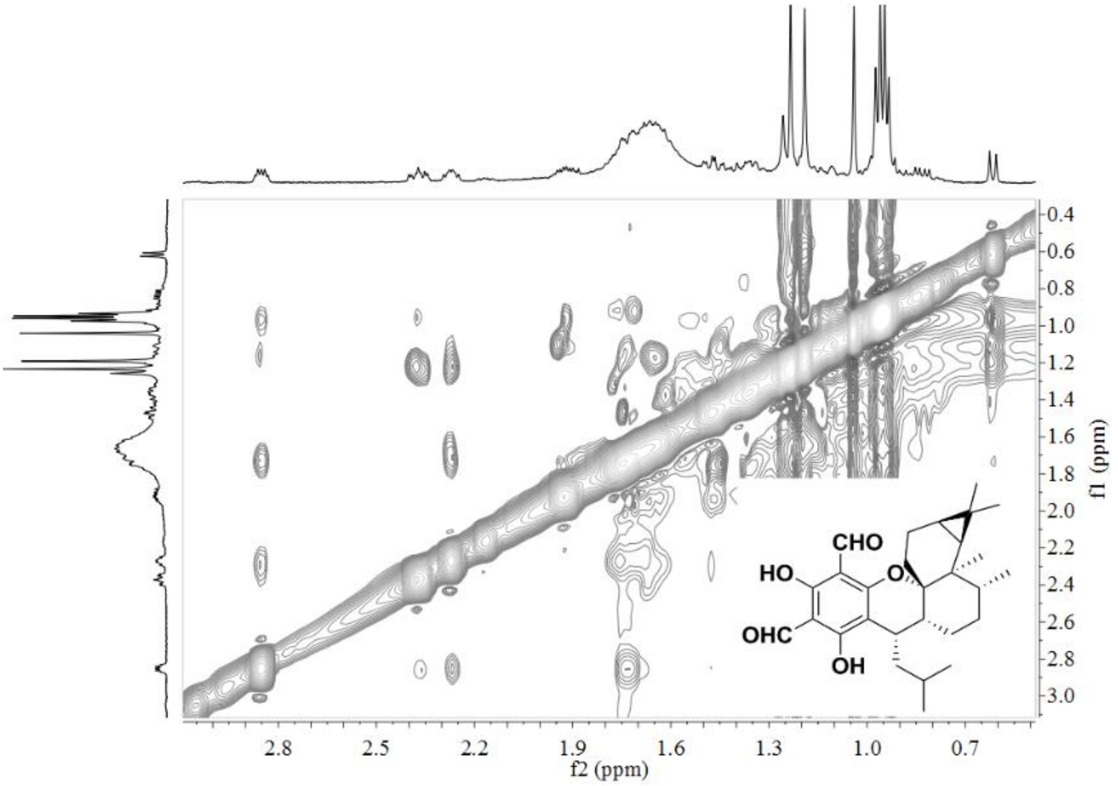

Figure S7-1 HRESIMS of eucalrobusone N (**5**) .

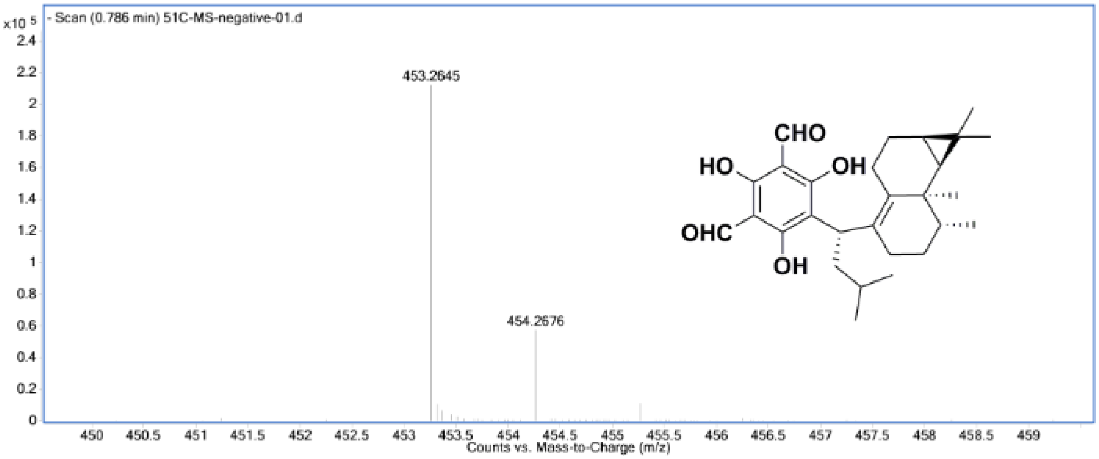

Elemental Composition Calculator

**Target m/z:**	453.2645	**Result type:**	Negative ions	**Species:**	[M-H]$^-$
**Elements:**		C (0-80); H (0-120); O (0-30); N(0-10); Cl (0-5)			
**Ion Formula**		**Calcalated m/z**		**PPM Error**	
C28H37O5		453.2646		0.38	

Figure S7-2 ^{1}H NMR spectrum of eucalrobusone N (**5**) in CD_3OD.

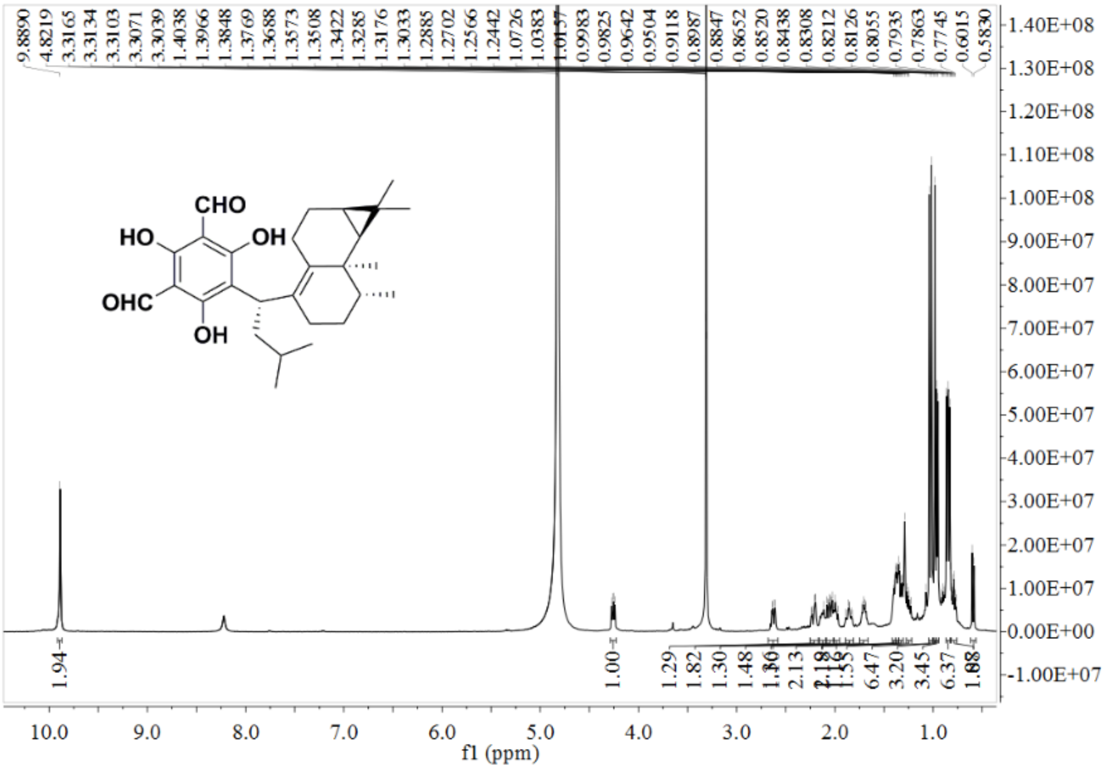

Figure S7-3 ^{13}C NMR spectrum of eucalrobusone N (**5**) in CD_3OD.

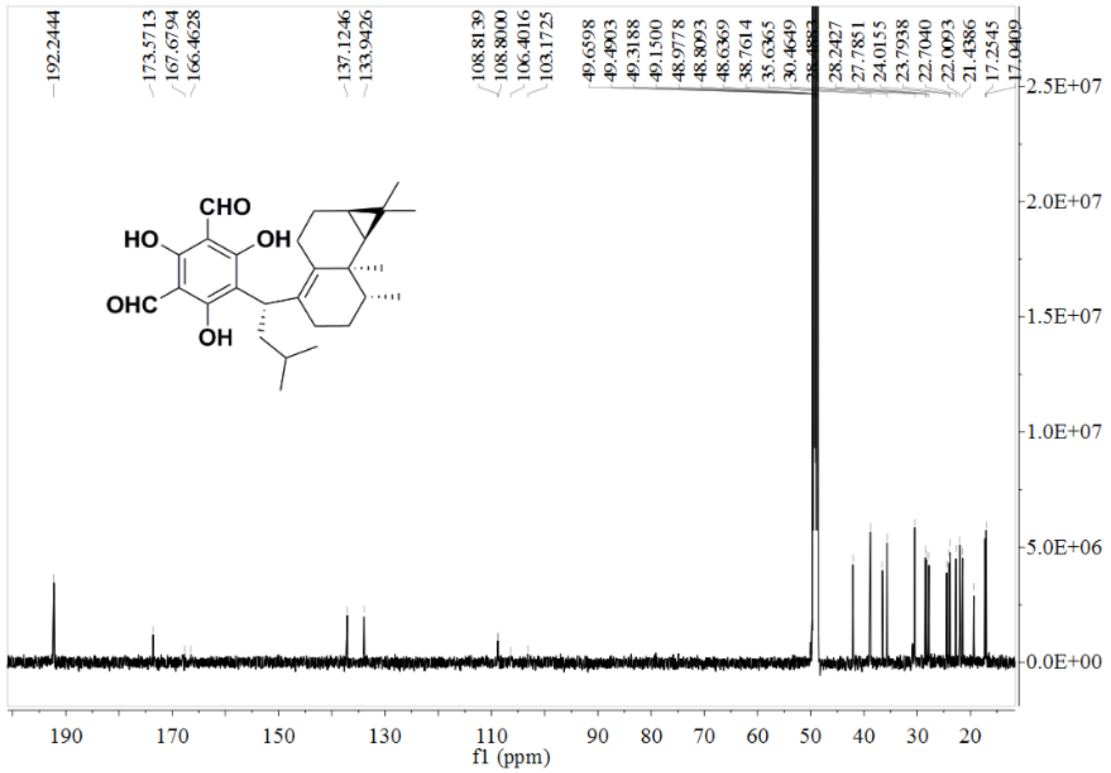

Figure S7-4 HSQC spectrum of eucalrobusone N (**5**) in CD_3OD.

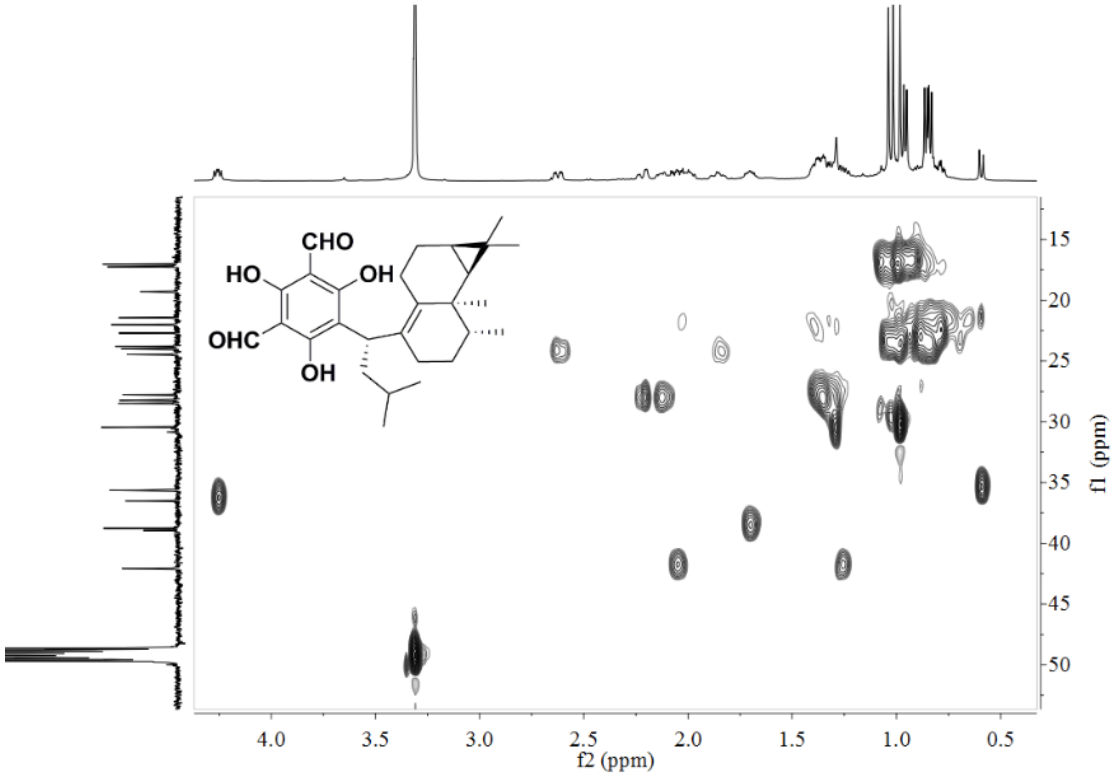

Figure S7-5 HMBC spectrum of eucalrobusone N (**5**) in CD_3OD.

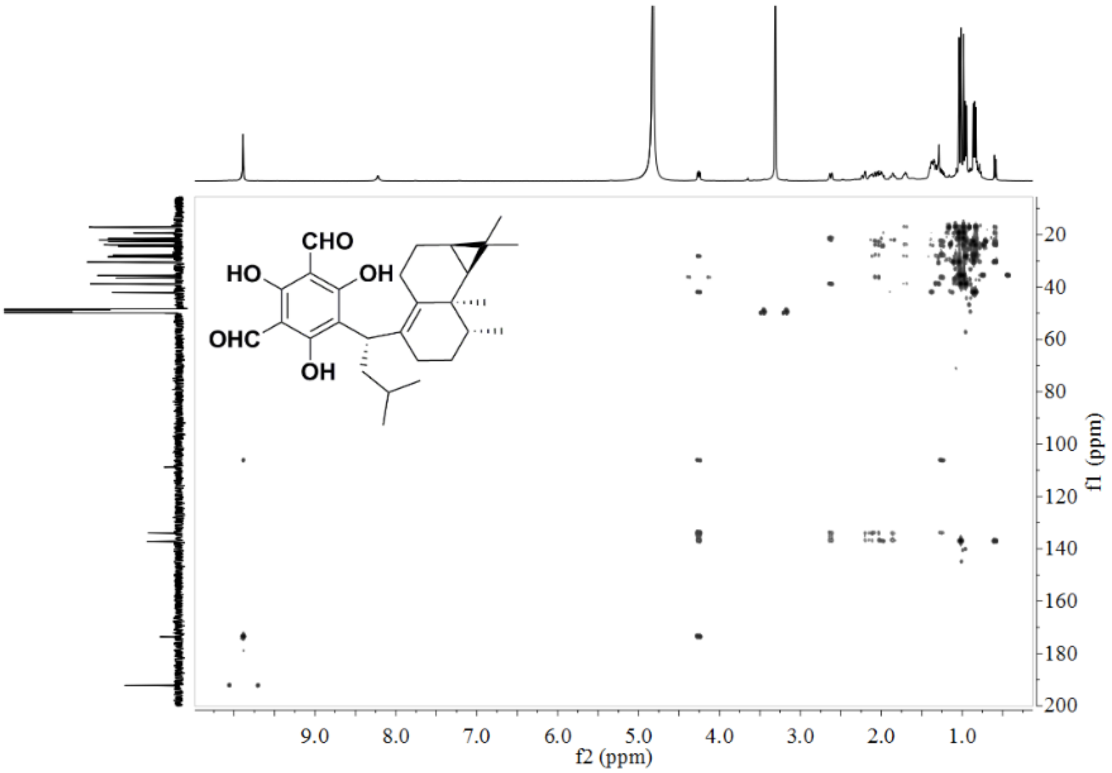

Figure S7-6 ROESY spectrum of eucalrobusone N (**5**) in CD_3OD.

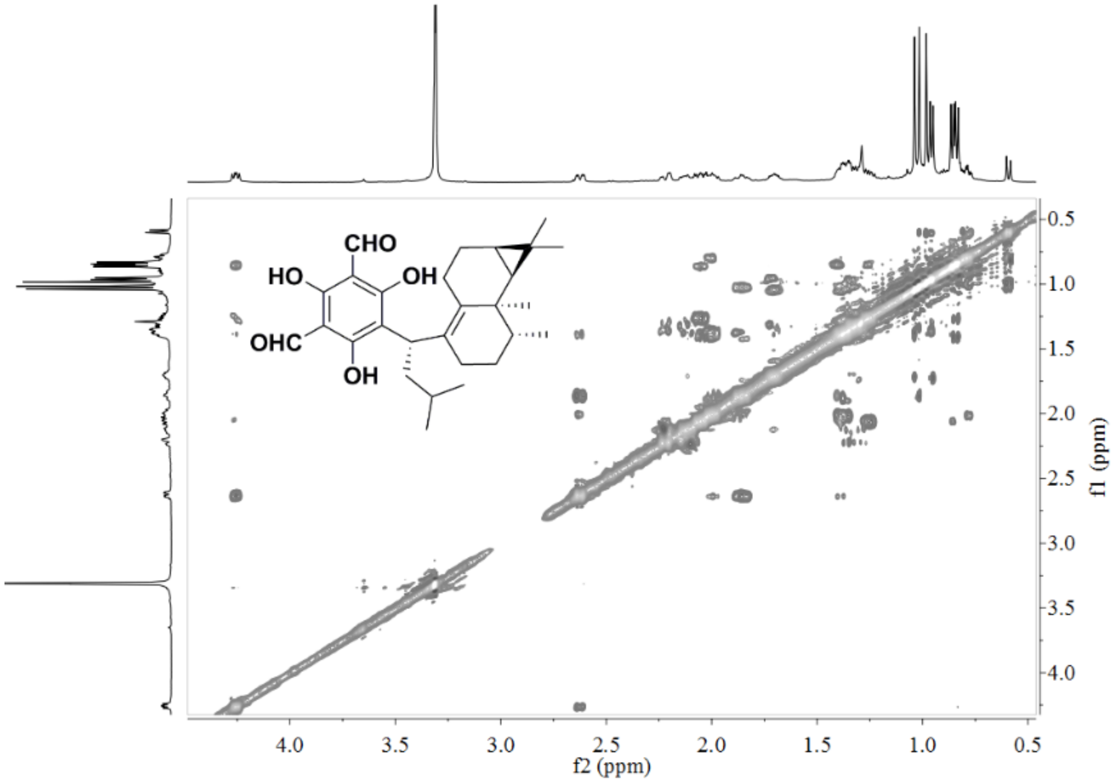

Figure S8-1 HRESIMS of eucalrobusone O (**6**) in $CDCl_3$.

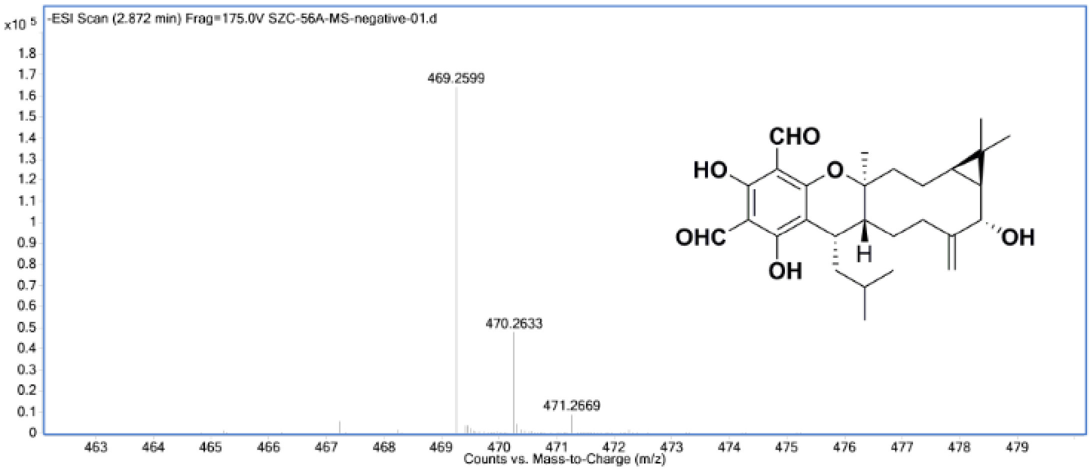

Elemental Composition Calculator

Target m/z:	469.2599	Result type:	Negative ions	Species:	[M-H]-
Elements:		C (0-80); H (0-120); O (0-30); N(0-10); Cl (0-5)			
Ion Formula		Calcalated m/z		PPM Error	
C28H37O6		469.2596		-0.73	

Figure S8-2 ^{1}H NMR spectrum of eucalrobusone O (**6**) in $CDCl_3$.

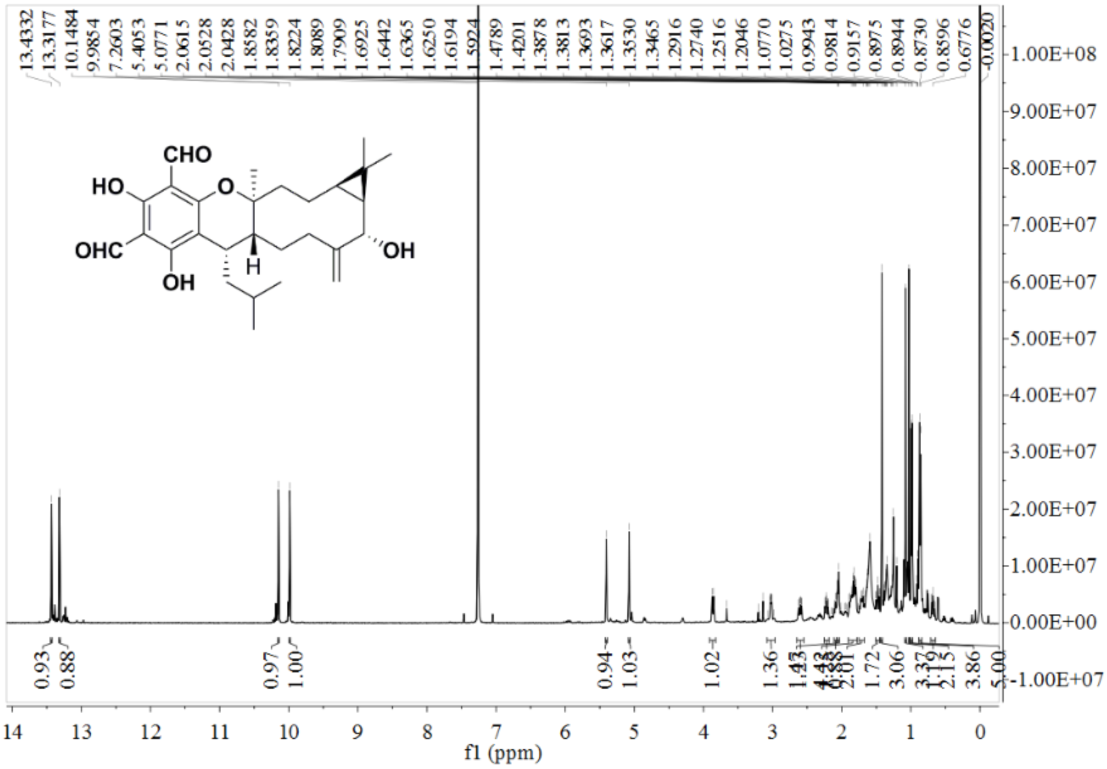

Figure S8-3 ^{13}C NMR spectrum of eucalrobusone O (**6**) in $CDCl_3$.

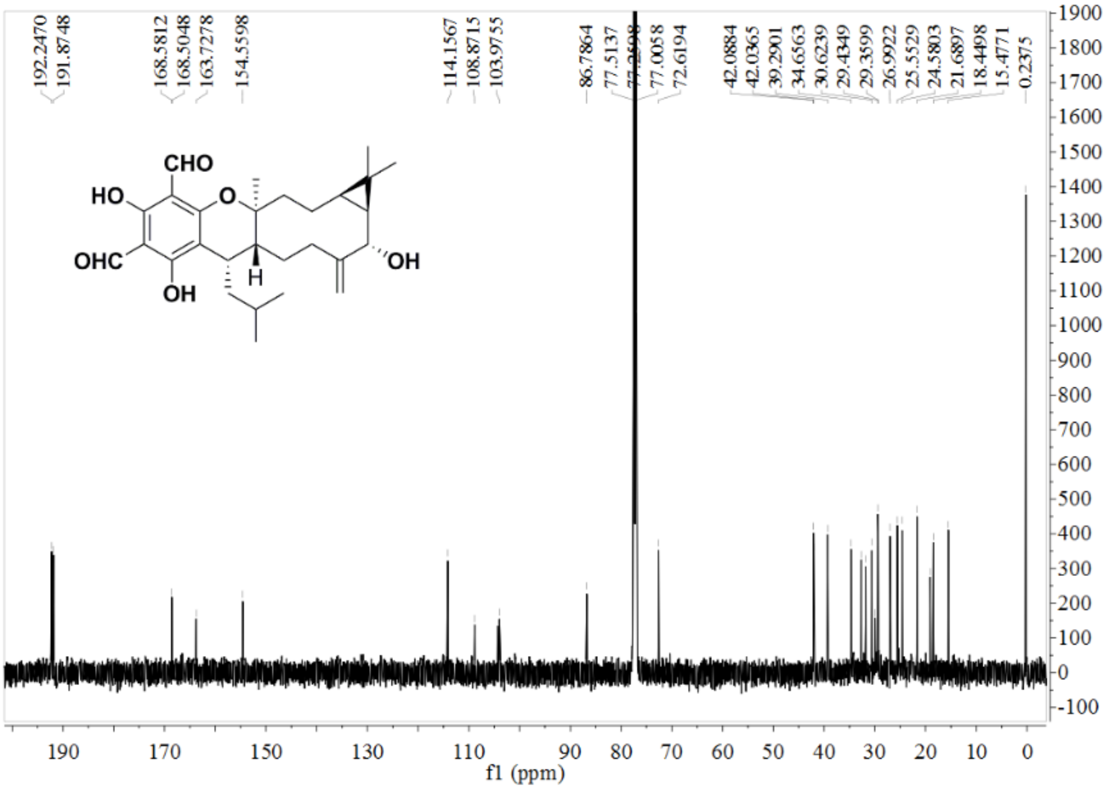

Figure S8-4 HSQC spectrum of eucalrobusone O (**6**) in $CDCl_3$.

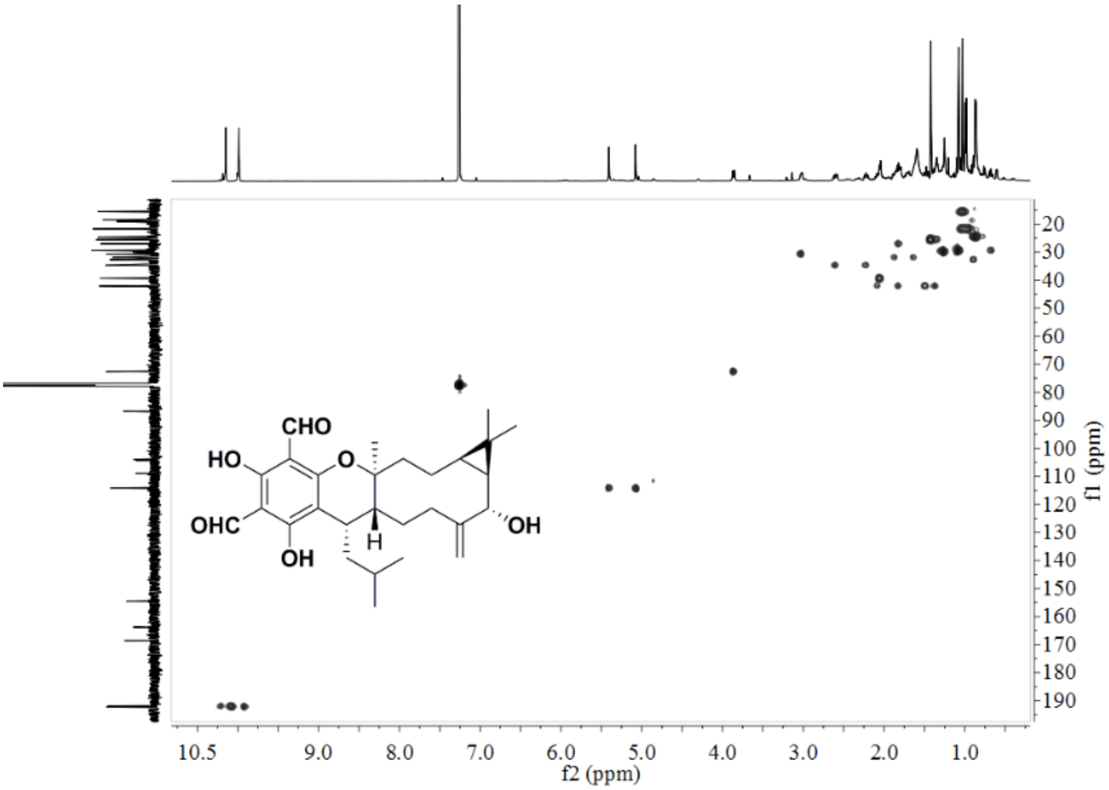

Figure S8-5 HMBC spectrum of eucalrobusone O (**6**) in $CDCl_3$.

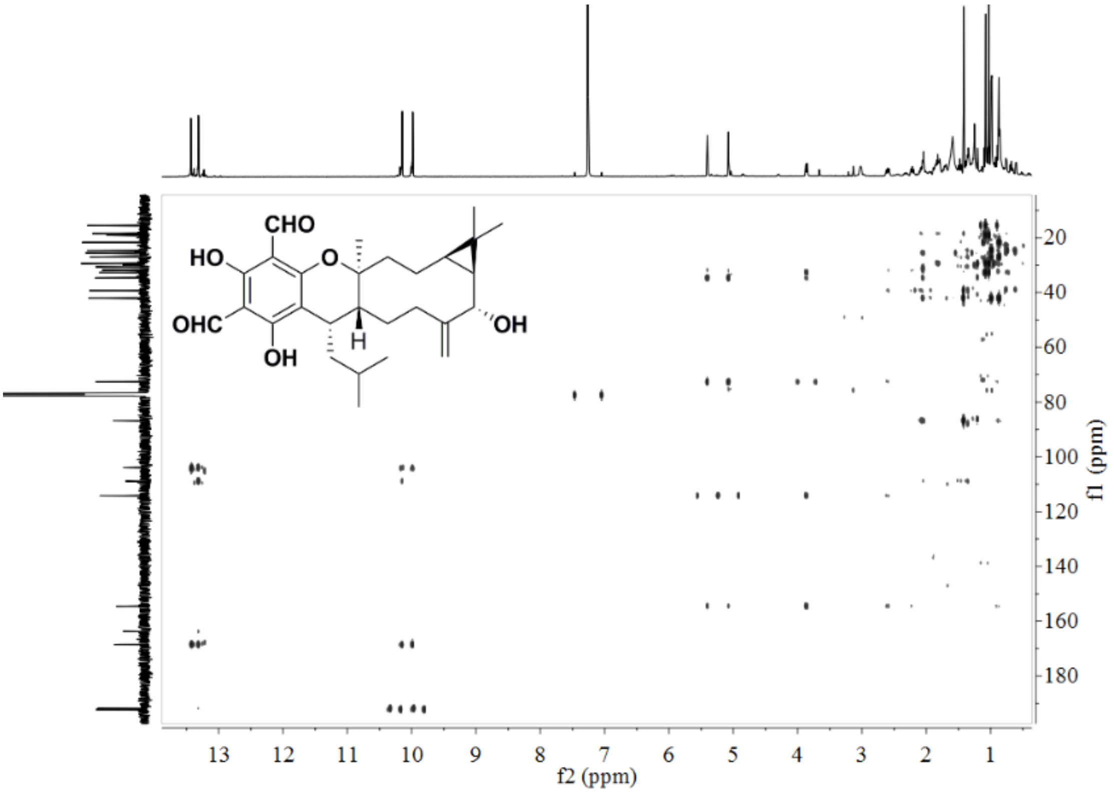

Figure S8-6 ROESY spectrum of eucalrobusone O (**6**) in $CDCl_3$.

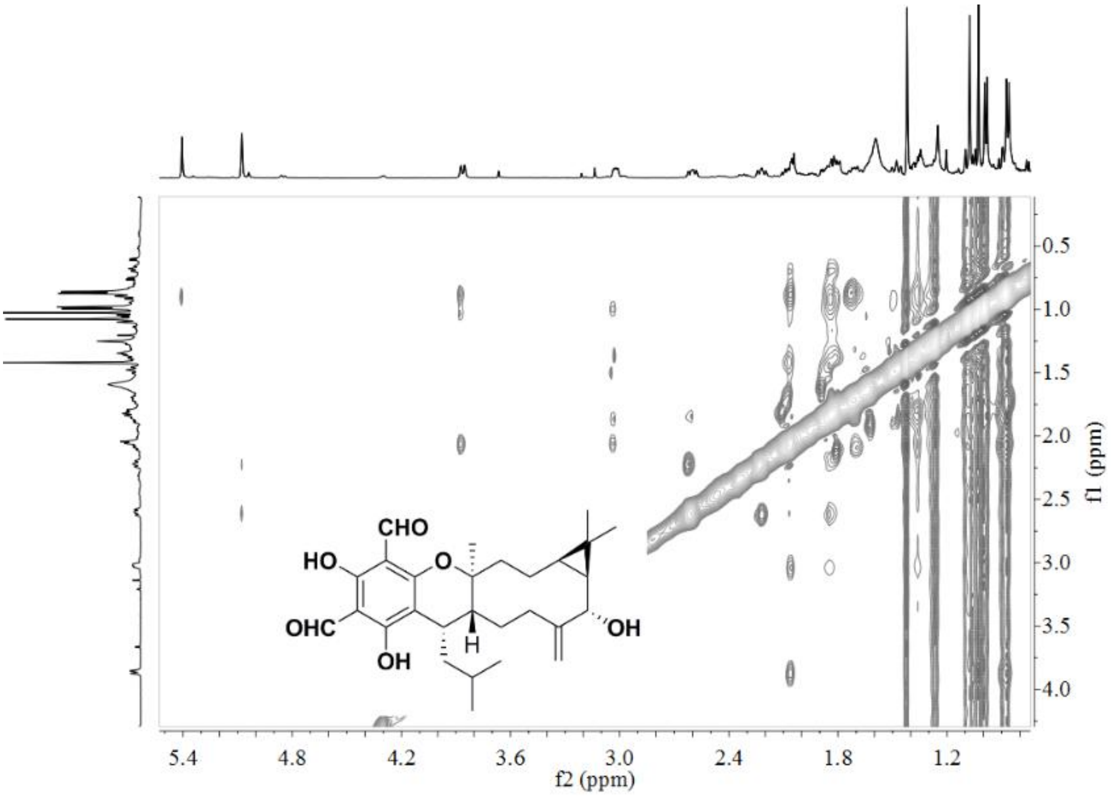

Figure S9-1 HRESIMS of eucalrobusone P (**7**) in $CDCl_3$.

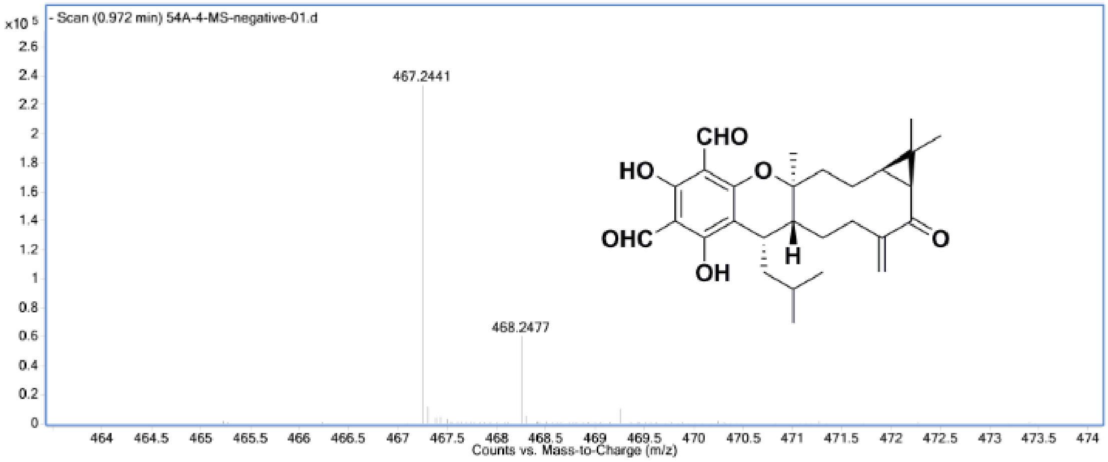

Elemental Composition Calculator

Target m/z:	467.2441	Result type:	Negative ions	Species:	[M-H]⁻
Elements:		C (0-80); H (0-120); O (0-30); N(0-10); Cl (0-5)			
Ion Formula		Calcalated m/z		PPM Error	
C28H35O6		467.2439		-0.33	

Figure S9-2 ^1H NMR spectrum of eucalrobusone P (**7**) in $CDCl_3$.

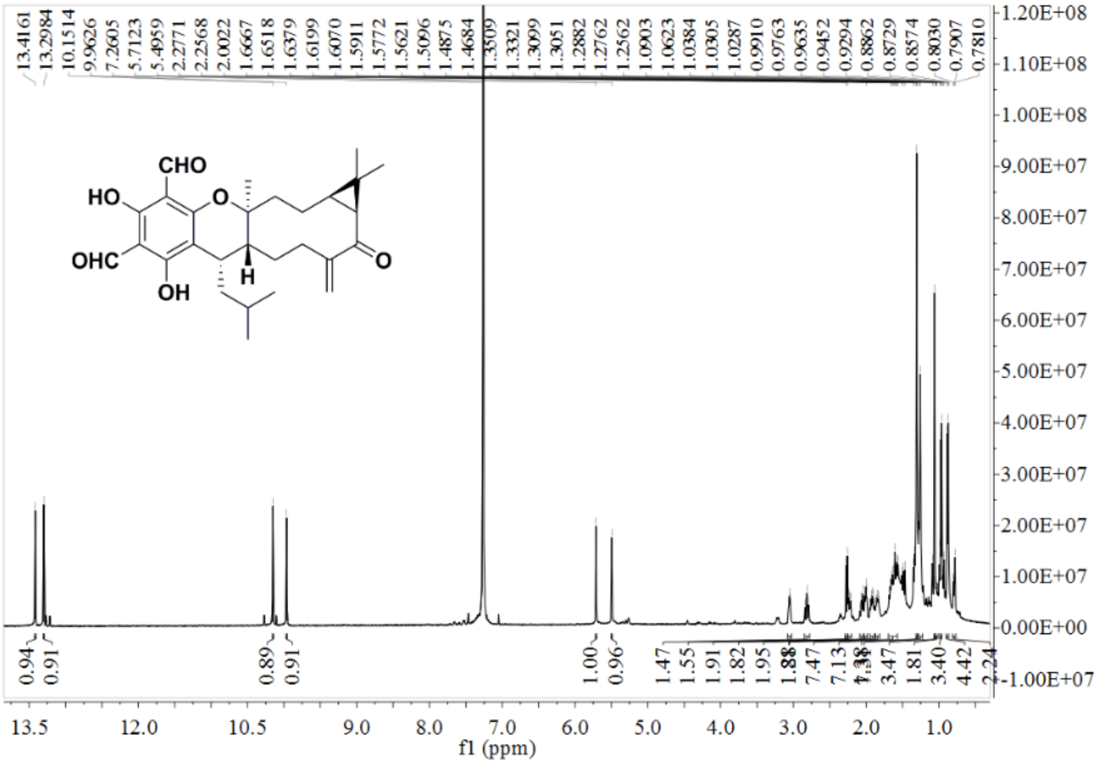

Figure S9-3 ^{13}C NMR spectrum of eucalrobusone P (**7**) in $CDCl_3$.

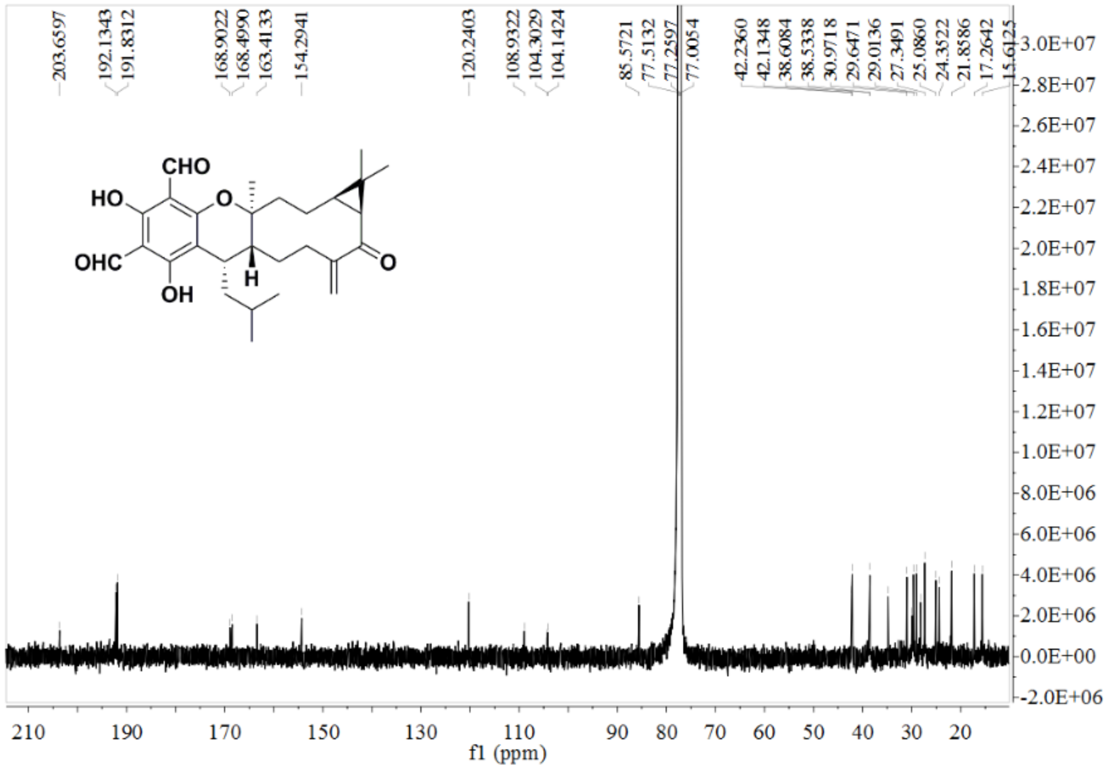

Figure S9-4 HSQC spectrum of eucalrobusone P (**7**) in $CDCl_3$.

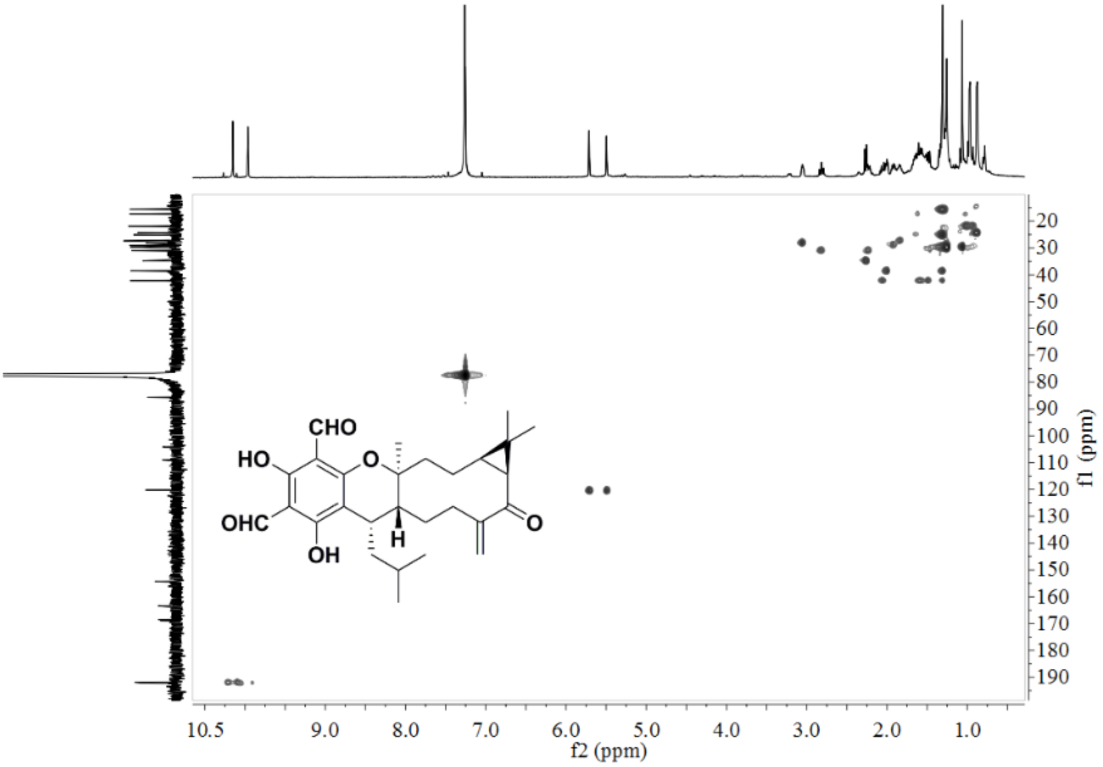

Figure S9-5 HMBC spectrum of eucalrobusone P (**7**) in $CDCl_3$.

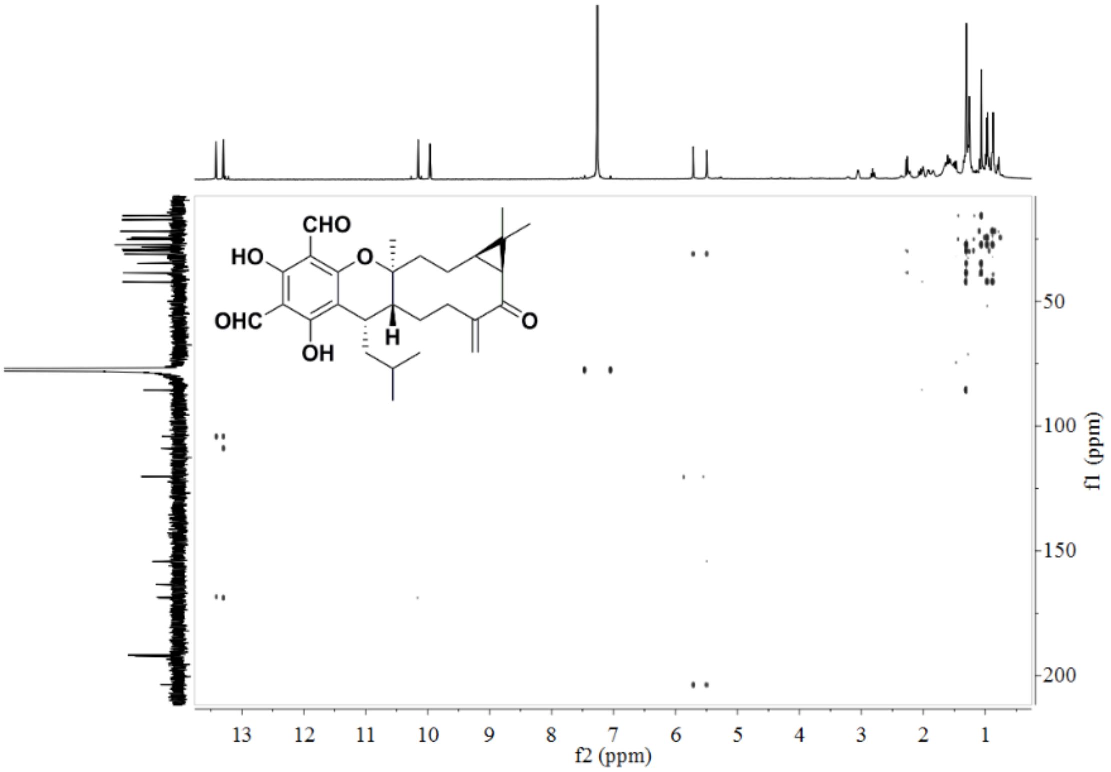

Figure S9-6 ROESY spectrum of eucalrobusone P (**7**) in $CDCl_3$.

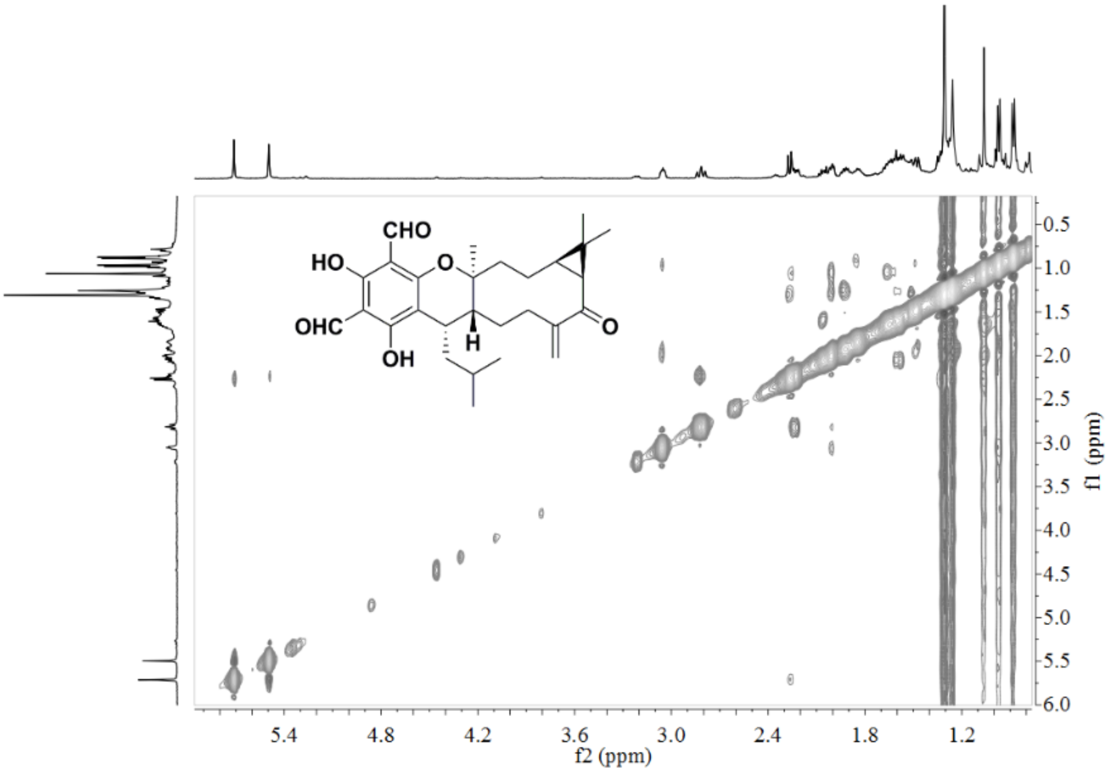

Supplement: Supporting Information [file srep39815-s1.pdf]
